# Bug22 influences cilium morphology and the post-translational modification of ciliary microtubules

**DOI:** 10.1242/bio.20146577

**Published:** 2014-01-07

**Authors:** Teresa Mendes Maia, Delphine Gogendeau, Carole Pennetier, Carsten Janke, Renata Basto

**Affiliations:** 1 Institut Curie, CNRS UMR144, 12 rue Lhomond, 75005 Paris, France; 2 Institut Curie, CNRS UMR 3306/INSERM U1005, Centre Universitaire, Bâtiment 110, 91405 Orsay, France

**Keywords:** Basal bodies, Cilia, Sperm individualization, Spermatogenesis, Tubulin post translation modifications

## Abstract

Cilia and flagella are organelles essential for motility and sensing of environmental stimuli. Depending on the cell type, cilia acquire a defined set of functions and, accordingly, are built with an appropriate length and molecular composition. Several ciliary proteins display a high degree of conservation throughout evolution and mutations in ciliary genes are associated with various diseases such as ciliopathies and infertility. Here, we describe the role of the highly conserved ciliary protein, Bug22, in *Drosophila*. Previous studies in unicellular organisms have shown that Bug22 is required for proper cilia function, but its exact role in ciliogenesis has not been investigated yet. Null *Bug22* mutant flies display cilia-associated phenotypes and nervous system defects. Furthermore, sperm differentiation is blocked at the individualization stage, due to impaired migration of the individualization machinery. Tubulin post-translational modifications (PTMs) such as polyglycylation, polyglutamylation or acetylation, are determinants of microtubule (MT) functions and stability in centrioles, cilia and neurons. We found defects in the timely incorporation of polyglycylation in sperm axonemal MTs of *Bug22* mutants. In addition, we found that depletion of human Bug22 in RPE1 cells resulted in the appearance of longer cilia and reduced axonemal polyglutamylation. Our work identifies Bug22 as a protein that plays a conserved role in the regulation of PTMs of the ciliary axoneme.

## Introduction

Cilia are specialized cell organelles that have motility and sensory functions. Their axoneme is built through nucleation of MTs from a basal body anchored at the plasma membrane, while assembly of the remaining cilia components normally relies on cargo transportation, in a process known as intraflagellar transport (IFT). Several ciliary proteins display a high degree of conservation, appearing widely present throughout eukaryotes. Mutations that perturb basal body anchoring, transition zone and cilium assembly, or the transport of specific signaling molecules to the cilium, are associated with a variety of diseases known as ciliopathies. These include several clinical manifestations such as retinal degeneration, polydactyl, kidney cysts, cranial malformations, mental retardation, obesity and sterility.

In the last decade several high throughput studies have contributed to the identification of cilia and centrosome components (Andersen et al., 2003; Avidor-Reiss et al., 2004; Keller et al., 2005; Li et al., 2004; Pazour et al., 2005; Stolc et al., 2005). One of these components is the Basal body up regulated gene 22 (Bug22), initially identified as a basal body component in the green algae *Chlamydomonas reinhardtii* (Keller et al., 2005). Bug22 is a remarkably conserved protein with homologs in all flagellated eukaryotes, but also in non-flagellated eukaryotes, being absent only from unicellular fungi and non-flagellated algae genomes (Broadhead et al., 2006). Bug22 is not exclusively associated with basal bodies, but also with cilia, in *Chlamydomonas*, *Tetrahymena*, *Paramecia*, mouse and human cells (Dawe et al., 2007; Pazour et al., 2005; Laligné et al., 2010; Ostrowski et al., 2002; Smith et al., 2005; Ishikawa et al., 2012).

In *Paramecium*, Bug22 is localised along the axonemes of motile cilia. Its depletion causes defects in ciliary morphology and motility without affecting overall axoneme structure (Laligné et al., 2010). Because functional analyses of Bug22 have only been performed in unicellular organisms (Hodges et al., 2011; Laligné et al., 2010), we decided to investigate its functions in a multicellular organism: the fruit fly *Drosophila melanogaster*. Here, we show that Bug22 in *Drosophila* associates with nucleus, basal bodies, sensory cilia and sperm flagella. Analysis of *Bug22* mutants revealed an uncoordinated phenotype, confirming a role for this protein in ciliogenesis. Unexpectedly, we have also found overly long basal bodies and defects in sperm individualization in *Bug22* mutants. Axonemal size control seems to be a general function of Bug22, as the depletion of the human homolog in RPE1 cells resulted in the formation of longer primary cilia. Interestingly both fly and human axonemes showed defects in the levels of tubulin post-translational modifications (PTMs) in the absence of Bug22. Our work suggests that Bug22 might play a conserved function in the regulation of axonemal size and functionality through the regulation of tubulin PTMs.

## Results

### *Drosophila* and human Bug22 are associated with cilia

**Fig. 1.**
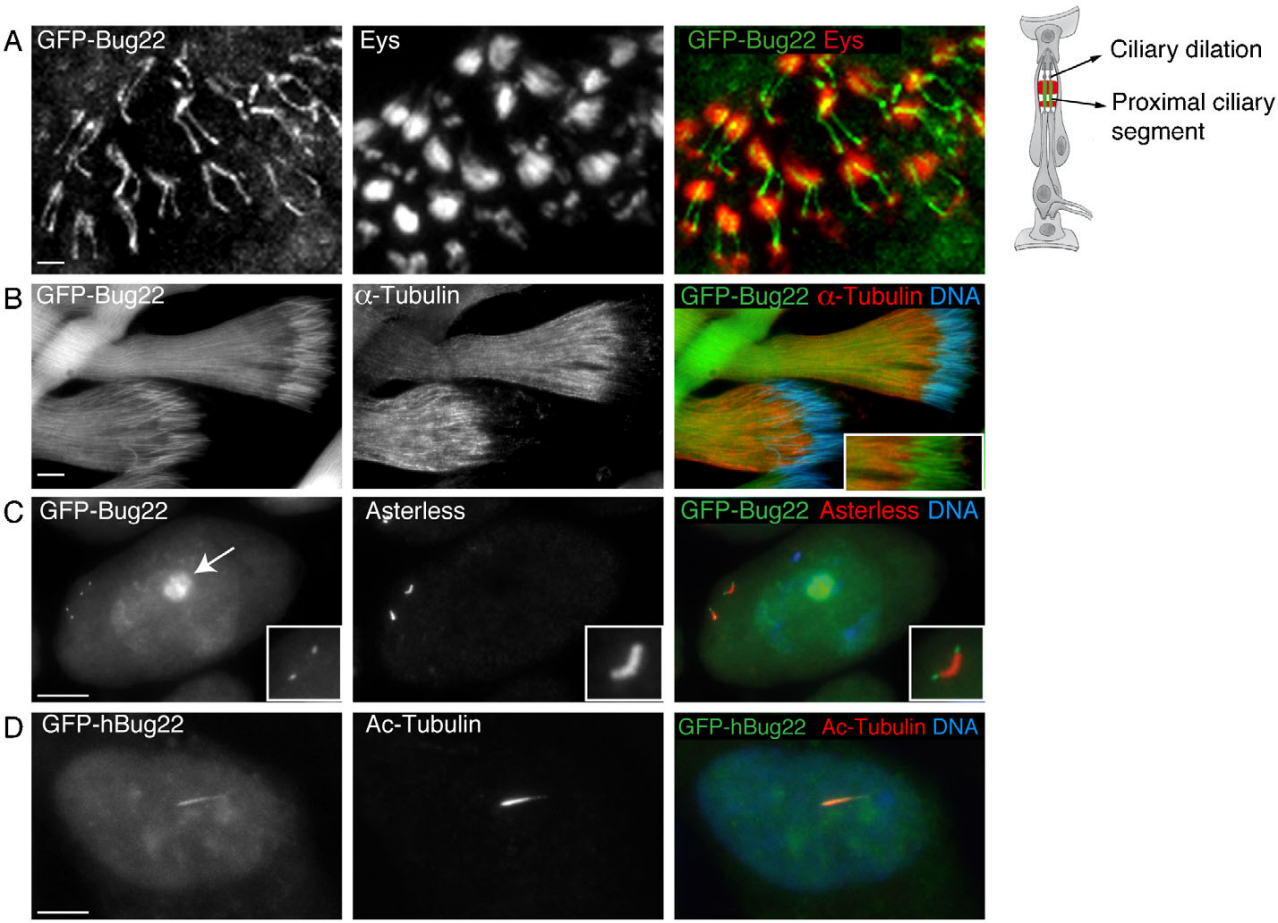
*Drosophila* and human Bug22 localise to the nucleus and cilia. (A) GFP-Bug22 (left and in green in the merged panel) localises to the cilia of antennal chordotonal organs. The proximal segments of the two cilia of each scolopale (sensory unit of the chordotonal organs, schematized on the right) can be identified by their position next to the Eys protein (middle panel, shown in red in the merged panel) labeling the scolopale extracellular space. Scale bar: 5 µm. (B) GFP-Bug22 (left and in green in the merged panel) localizes along the entire length of the sperm flagella and the nucleus. α-tubulin (middle panel is shown in red in the merged panel) and DNA is in blue. The inset in the merged panel shows a higher magnification view of the sperm nuclei region with α-tubulin and GFP-Bug22 to illustrate the nuclear localisation of Bug22. Scale bar: 5 µm. (C) Primary spermatocyte expressing GFP-Bug22 (left and in green in the merged panel) and stained for the centriole marker Asterless (Asl) that labels the entire centriole at this stage (middle and shown in red in the merged panel) and for DNA (shown in blue in the merged panel). GFP-Bug22 localises to the distal segment of the giant centrioles, a region that is not labeled by the centriole marker Asl and that is composed of doublets of MTs (Carvalho-Santos et al., 2012; Riparbelli et al., 2013; Riparbelli et al., 2012; Tates, 1971). In addition GFP-Bug22 also localizes to the nucleus and it is enriched in the nucleolus (arrow in the left panel). Scale bar: 10 µm. (D) hTERT-RPE1 cells transfected with GFP-hBug22 (left, in green in the merged panel) and stained for Ac-Tubulin to label the primary cilium (shown in red in the merged panel) and DNA (shown in blue in the merged panel). Scale bar: 3 µm.

In *Drosophila*, Bug22 is encoded by the *CG5343* gene, which codes for a protein with an estimated mass of ∼23 kDa. In order to study its localisation, we raised antibodies and generated constructs to express GFP-tagged versions of full length Bug22. We were unable to obtain any specific immunoreactivity from sera of animals immunised with either human or *Drosophila* Bug22, probably due to its high conservation and thus poor antigenicity. A commercially available antibody (GTL-3, see Materials and Methods) specifically recognised Bug22 on western blots (see below) but not in immunostainings. Hence, analysis of Bug22 in flies was based on transgenic lines that express GFP-Bug22 under a ubiquitous promoter, termed Ubq (Basto et al., 2008; Peel et al., 2007). We found that GFP-Bug22 localised to cilia of chordotonal organs in sensory neurons localised in the fly antenna (Fig. 1A) and was also associated with the sperm flagellum (Fig. 1B). While analyzing the male testis, we also noticed a clear signal of GFP-Bug22 at the tip of the giant centrioles of primary spermatocytes (Fig. 1C). This localization was more distal than that of other known centriole proteins such as Asterless (Asl) (Fig. 1C) or Sas4 and PACT (data not shown). Ultrastructural analysis of these centrioles has shown their distal-most segment actually corresponds to a small primary cilium, composed of a transition zone and a short axoneme (Carvalho-Santos et al., 2012; Riparbelli et al., 2012; Tates, 1971) and so we conclude that GFP-Bug22 is associated with this primary cilium. In sensory neurons and in sperm cells, Bug22 appeared localized to the nucleus (data not shown for sensory neurons, Fig. 1B-inset). In primary spermatocytes, Bug22 in addition to the nucleus appeared strongly enriched at the nucleolus (Fig. 1C, arrow). Importantly, we have never seen Bug22 associated with centrosomes or basal bodies in other cell types (data not shown).

We finally confirmed that, as described previously (Ishikawa et al., 2012), human Bug22 (hBug22) is also ciliary. In hTERT-RPE1, GFP-hBug22 distributes along the length of primary cilia and the nucleus (Fig. 1D). Thus, Bug22 is a conserved ciliary protein.

### Bug22 plays essential roles in ciliated and non-ciliated tissues in *Drosophila*

**Fig. 2.**
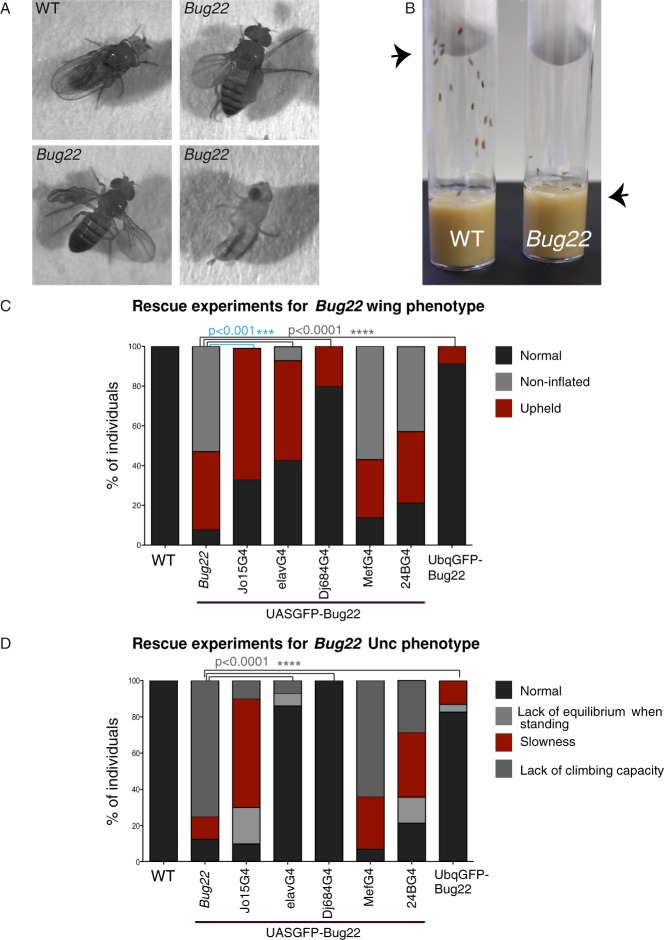
Characterisation of *Bug22* flies. (A) Images from WT and *Bug22* flies. From left to right: a WT adult fly at rest; a *Bug22* fly, showing an abnormal positioning of its wings; a *Bug22* fly, presenting an improperly unfolded wing (right); a *Bug22* fly, showing severe morphological defects in its wings and legs and so it cannot stand in an upright position. (B) Image from vials containing WT (left) and *Bug22* (right) flies. While the majority of WT flies rapidly migrate to the top (arrowhead) of the vial after this has been tapped, *Bug22* mutants remain at the bottom of the tube (arrowhead). (C,D) Graphs representing rescue experiments for *Bug22* phenotypes of wing posture/inflation and climbing capacity. Percentage of flies belonging to various phenotypic classes is represented. At least 25 flies from each genotype were scored. Chi-square tests were used to assess statistical differences between the mutant and the rescue experiments considering the “Normal” phenotype. Only statistical significances are shown. JO15-Gal4 driver was used for chordotonal organ expression, which contain ciliated neurons. elav-Gal4 and DJ6884-Gal4 for neuronal expression, 24B-Gal4 and Mef2-Gal4 for mesoderm and muscle expression. Ubq promoter drives constitutive transgenic expression.

To analyse the function of Bug22 in flies we generated a null allele for *Bug22* by homologous recombination (supplementary material Fig. S1A). We obtained one single allele (supplementary material Fig. S1B) that will be referred to as *Bug22*. Importantly GFP-Bug22 transgenes rescued this mutation (Fig. 2C,D), showing that the phenotypes described below are solely due to the loss of Bug22.

*Bug22* flies were viable and could develop until late pupal stages. However, eclosed adult flies presented reduced lifespan, which could vary from a few hours to up to a few days. Furthermore, and consistently with the suspected functions of Bug22 in cilia, about half of the mutants displayed an uncoordinated phenotype (here referred to as Unc) similar to *Drosophila* mutants with defects in ciliogenesis (Baker et al., 2004; Martinez-Campos et al., 2004; Basto et al., 2006; Enjolras et al., 2012). Bug22 flies were morphologically normal, but presented defects in the positioning of their wings (Fig. 2A) and, despite being able to walk, did not present the same feeding and foraging behaviour as wild-type (WT) flies. Indeed, *Bug22* flies were sedentary and showed strongly diminished climbing activity (Fig. 2B), similarly to gravitaxis mutants that have impaired mechanosensation in chordotonal organs (Chatterjee et al., 2011; Gong et al., 2004; Texada et al., 2008). In addition to these phenotypes, *Bug22* males only produced immotile sperm. The remaining adult mutant progeny, however, presented more severe phenotypes. These included defects in wing inflation (unfolding), a “slimy” body wall and incapacity to stand in an upright position (Fig. 2A), which altogether resulted in the death of the mutant flies only a few hours after eclosion. Thus, *Bug22* flies fall on two distinct phenotypic classes: one that displays an Unc phenotype and a second one with even more severe phenotypes.

To better understand the reasons for these differences, we decided to investigate the nature of the defects found in *Bug22* mutants. We used the UAS/Gal4 system to express a UAS-*Bug22* construct in different cell types in the *Bug22* background. Expression of *Bug22* exclusively in the mesoderm (Mef2-Gal4, 24B-Gal4), which has been shown to restore climbing activity and wing posture in mutants presenting mitochondrial defects affecting the skeletal muscle system (Greene et al., 2003), did not rescue any trait of *Bug22* flies (Fig. 2C,D). Using a chordotonal-organ-specific Gal4 driver (JO15-Gal4) (Sharma et al., 2002), we obtained only a partial rescue of *Bug22* phenotypes as some of these flies still presented defects in locomotion and climbing activity (Fig. 2C,D). Strikingly, an almost complete rescue of all defects, including body morphology (non-inflated wings and “slimy” body wall), locomotion, climbing activity and lifespan was obtained when we used a pan-neuronal (elav-Gal4) (Luo et al., 1994) or adult nervous system (DJ684-Gal4) (Seroude et al., 2002) Gal4 drivers (Fig. 2C,D). These results lead us to conclude that Bug22 functions in both ciliated and non-ciliated neurons. Importantly, ubiquitous expression of Bug22 using a ubiquitous promoter (pUbq) largely rescued the wing, Unc and sperm immotility phenotypes (Fig. 2C,D). Overall, our analysis shows that *Drosophila* Bug22 plays essential functions in ciliated cells: sensory neurons and sperm, and suggests that Bug22 has additional functions in the nervous system.

### Bug22 is required for differentiation of the sperm flagellum

**Fig. 3.**
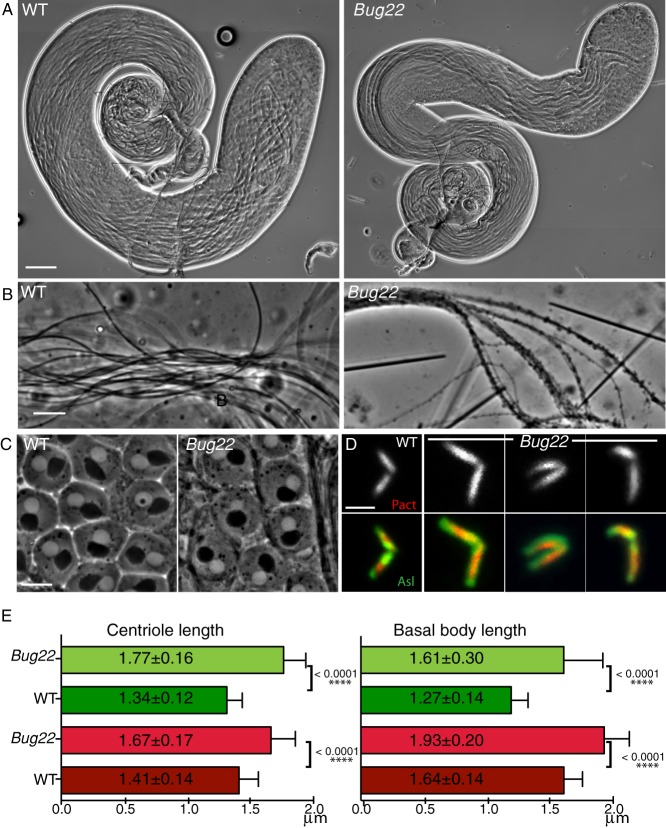
Characterisation of *Bug22* testes. (A) Pictures from adult WT (left) and *Bug22* testes (right) showing regular testes morphology. Scale bar: 100 µm. (B) High magnification pictures of WT (left) and *Bug22* (right) spermatids showing the presence of thicker sperm tails in the mutant. Scale bar: 10 µm. (C) Phase contrast image showing that *Bug22* (right) onion stage spermatids are indistinguishable from WT (left). Every post-meiotic round spermatid contains one nucleus (white circle) adjacent to one nebenkern (black circle), both structures having approximately the same size. Onion stages from at least 20 males were analysed. Scale bar: 10 µm. (D) Images of WT and *Bug22* dividing primary spermatocyte centrioles expressing RFP-PACT (top panel and shown in red in the merged panel) and Asl (shown in green in the merged panel). Scale bar: 2 µm. (E) Graphs showing measurements of centriole (left) and basal body (right) lengths in WT and *Bug22*. Measurements were based on the fluorescence signal of the transgenic protein RFP-PACT (red bars) and Asl (green bars). Mean values of length (±) represent the standard deviation from more than 50 centrioles from meiosis I or II spermatocytes (left) and elongated spermatids (right). Student's t-tests were performed to assess statistical differences.

To understand the requirements for Bug22 in male fertility, we analysed the genital tracts of *Bug22* males and found defects in spermatogenesis. Indeed, although *Bug22* male reproductive system appeared morphologically normal (Fig. 3A), it contained empty seminal vesicles (not shown). Furthermore, under the light microscope various defects at the level of elongated spermatid flagella could be perceived: these were immotile, appeared much less flexible than the WT and finally, the flagella appeared thicker and presented cytoplasmic bulges (Fig. 3B).

We had observed that GFP-Bug22 localised to the distal tip of spermatocyte centrioles. Defects in meiosis are frequently present in centriole or centrosome mutants (Basto et al., 2006; Martinez-Campos et al., 2004; Mottier-Pavie and Megraw, 2009; Rodrigues-Martins et al., 2007). Characterisation of meiotic divisions showed that spindle poles and spindle morphology appeared normal and we never detected chromosome segregation defects (data not shown). Further inspection of the ploidy and number/size of nebenkerns (mitochondrial derivatives) in post-meiotic cells (onion stage spermatids) by phase contrast microscopy confirmed the absence of meiotic defects in *Bug22* (Fig. 3C). Strikingly, we noticed that *Bug22* primary spermatocyte centrioles were longer than their WT counterparts and assembled centriole pairs with strange bends and/or arrangement (Fig. 3D,E). This difference in length was still observed at later stages of spermatogenesis, when these centrioles behave as basal bodies to nucleate sperm flagella (Fig. 3E). Note that the extent of the signal occupied by Asl decreases in basal bodies while PACT continues to increase. This is in agreement with the characterization of Asl localization to the a proximal centriole-like structure after meiosis (Blachon et al., 2009).

**Fig. 4.**
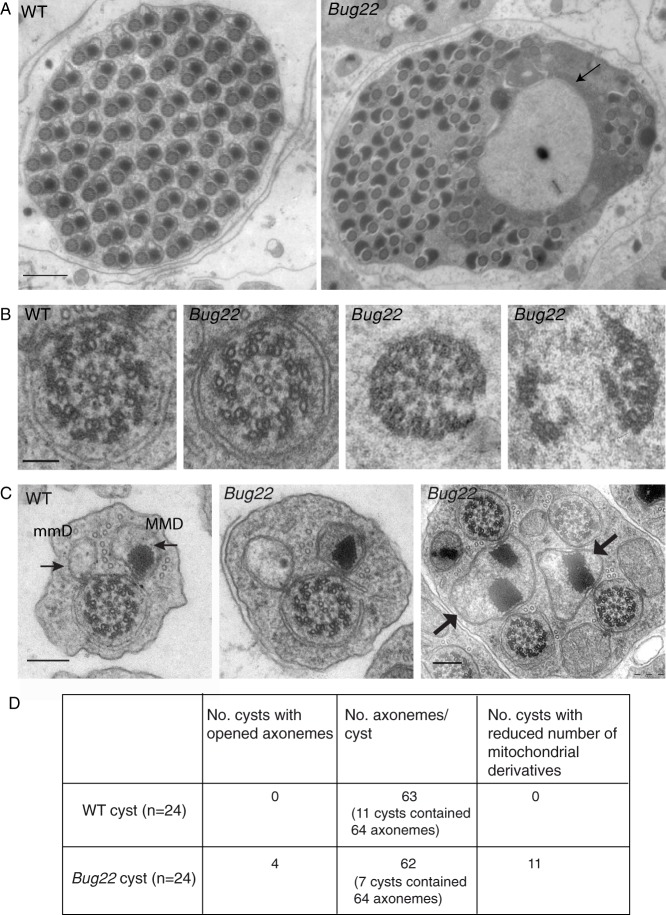
TEM analysis of *Bug22* testes. (A) TEM micrographs of cross sections of WT (left) and *Bug22* (right) post-individualized cysts. In the WT cyst a highly ordered arrangement of sperm tails axonemes surrounded by membrane can be seen, while the *Bug22* mature cysts at a comparable stage of differentiation present a high level of disorganization with most axonemes appearing un-individualized. Large membrane delimited non-electron dense inclusion bodies can occasionally be seen in non-individualized cysts (arrow). Scale bar: 1 µm. (B) TEM micrographs from cross-sections of sperm flagella from WT and *Bug22* spermatid cysts. In both genotypes, axonemes form correctly, displaying a 9+9+2 MT arrangement. However, in a few post-elongation cysts abnormal axonemes (right) were found that appeared either slightly opened or completely disassembled. Scale bar: 0.2 µm. (C) Representative spermatid tails from WT (left) and *Bug22* (right) testes. In both genotypes a major (MMD) and a minor (mmD) mitochondrial derivative (arrows) can be seen associated with each axoneme. In the mutant spermatid tails, the appearance of two axonemes sharing one major mitochondrial derivative was frequently seen (arrows) in late-elongation or individualizing cysts. Scale bars: 0.2 µm. (D) Quantification of defects in *Bug22* spermatid cysts analysed by TEM.

To further investigate *Bug22* defects in spermatogenesis, we analysed the ultrastructure of the sperm axoneme by transmission electron microscopy (TEM). While WT cysts appeared well organised, every *Bug22* post-elongating cyst was very disorganised and contained large amounts of cytoplasm (Fig. 4A), which was never observed in the WT. In addition, in *Bug22* cysts large membrane delimited non-electron dense inclusion bodies (never seen in the WT) could be seen (Fig. 4A, arrow). Cross-section analysis of control and *Bug22* sperm tails from pupal and adult stages showed that axonemes were well assembled (Fig. 4A,B). Axoneme features such as the 9+9+2 MT doublets, radial spokes and the sets of dynein arms were always present and did not present any obvious defects (Fig. 4B; data not shown). Of notice, however, while examining adult testes, we found that 16.8% of the cysts (*n* = 4/24) contained axonemes that were disrupted, appearing slightly or completely opened (Fig. 4B,D). Since this type of defect was infrequent and never observed in testes taken from pupal stages, we interpret them as resulting from a failure in the maintenance of the axoneme structure, rather than defects in the assembly process.

While *Bug22* sperm tails appeared morphologically normal in most cases, we also observed defects in mitochondria derivatives (Fig. 4C, arrows). Normally, a triad of one axoneme, one major and one minor mitochondrial derivative can be seen. In mutant tails, however, a high portion of cysts (45.6%, *n* = 24) contained a few sperm tails with mitochondrial derivatives in insufficient number, a defect that was not associated with meiotic defects.

In *Drosophila*, at the end of spermatogenesis, a process known as sperm individualization generates 64 mature and individualized sperm cells within a cyst. Individualization requires the movement of multiprotein complexes, called individualization complexes (ICs), along the length of sperm tails. Individualization results in the reduction of spermatid cell volume, through the expulsion of large masses of cytoplasm and organelles, dispensable for individual sperm cell function as ICs migrate (Tokuyasu et al., 1972a). We conclude that Bug22 is required for sperm individualization and thus maturation.

### Bug22 is required for the migration of individualization complexes

**Fig. 5.**
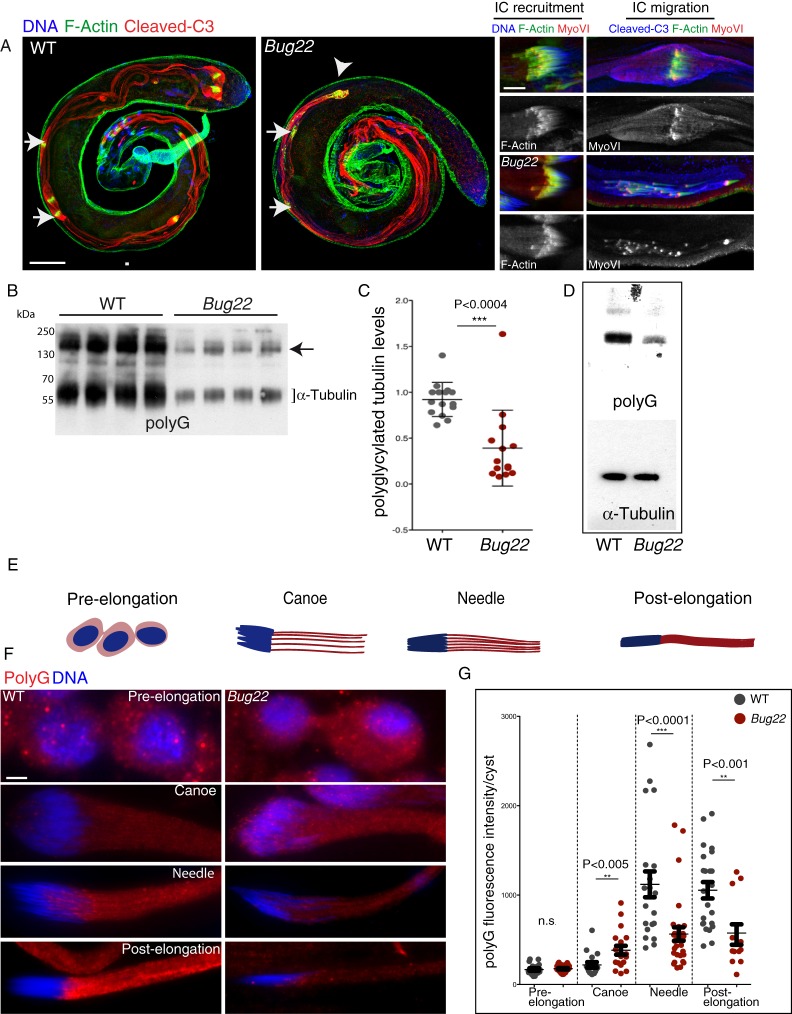
Analysis of sperm individualization and tubulin modifications in WT and *Bug22* testes. (A) Left – immunostaining of whole mount WT and *Bug22* testes stained for F-actin (shown in green), cleaved caspase3 (Cleaved-C3, shown in red) and DNA (shown in blue). In the WT panel individualizing cysts can be observed with migrating ICs (arrows) and waste bags at the extremity (arrowheads). In *Bug22*, the ICs (arrows) lagged and the only waste bag detected (arrowheads) appeared very abnormal and reduced in size. Right – higher magnifications of WT (top) and *Bug22* (bottom) sperm tails undergoing individualization. F-actin is shown in green, MyoVI is shown in red and DNA/Cleaved-C3 in blue. ICs recruitment to the sperm nuclei occurs similarly to WT (top and bottom left), but the IC migration (top and bottom right) was not synchronous. MyoVI localisation did not seem to be perturbed in *Bug22* testes. Scale bars: (left) 100 µm, (right) 10 µm. (B) Immunoblots from WT and *Bug22* testes extracts probed with polyG antibodies that recognise polyglycylation. The bracket indicates the tubulin region and the arrow points to a higher molecular weight band corresponding to unidentified polyglycylated proteins that are also reduced in the mutant. (C) Quantification of tubulin polyglycylation levels detected by western blot in WT and *Bug22* single testes. Values shown are relative to a WT sample set as reference within each of four experiments performed, and had been previously normalized to a loading control. The lines show the mean value ± SD. A two-tailed unpaired t-test was used to assess statistical differences between polyG levels in WT and mutant. (D) Immunoblots from WT and *Bug22* testes extracts probed with polyG (that recognise polyglycylation) and α-tubulin antibodies. Both lanes contain equivalent protein amounts determined by Coomassie staining. This result shows that in the mutant, tubulin levels are similar to WT. (E) Definition of the four stages of spermatid differentiation. Nuclei are shown in blue and sperm tails in red. (F) Representative images of WT and *Bug22* cysts, stained with polyG antibodies (to reveal polyglycylation, shown in red) and for DNA (shown in blue), during spermatid differentiation. Scale bar: 5 µm. (G) Plots showing polyG fluorescent intensity measurements in WT (gray) and *Bug22* mutant cysts (red). The median intensities of all cysts analysed (each dot corresponds to a different cyst) ± SD are shown. A two-tailed unpaired t-test was used to assess statistical differences between WT and mutant at each stage. n.s. stands for no statistical significance. Cysts were classified according to their differentiation stage defined in E. Polyglycylation of WT sperm tails occurs at a defined developmental timing during spermatid differentiation. Such defined program appears altered in *Bug22* sperm tails from canoe stages onwards.

Sperm individualization starts with the assembly of ICs around each of the elongated nuclei. ICs are composed, among many other components of F-actin structures, called actin cones, which have been well characterised (Noguchi and Miller, 2003). Subsequently, the ICs begin to move along the sperm tail. Because this migration is accompanied by the continuous accumulation of extruded cell material around the IC, a voluminous structure called the cystic bulge (CB) is created at this stage (Fig. 5A, arrows). Finally, when the complex reaches the end, of the now individualized tails, the CB turns into a waste bag (Fig. 5A, arrowheads), which is eventually degraded. In *Bug22* flies, just like in WT, ICs were correctly assembled at the spermatid nuclei (Fig. 5A, insets). Moreover, the ICs moved and migrated away from the nuclei (Fig. 5A, arrows and insets). However, unlike in WT individualizing cysts, the ICs appeared dispersed and somehow lagged along the sperm tails (Fig. 5A, arrows and insets). Asynchronous movement of actin cones is commonly observed in *Drosophila* mutants with defective sperm individualization (Fabrizio et al., 1998; Riparbelli and Callaini, 2007; Zhou et al., 2011). Accordingly, CBs and waste bags in *Bug22* appeared smaller and contained less material than in WT (Fig. 5A, arrowheads). Activation of caspases in a spatially regulated manner is also required during sperm individualization, to promote the formation of the CB (Arama et al., 2003; Kaplan et al., 2010), but we did not observe any differences between WT and *Bug22* cysts. We concluded that in the absence of Bug22, ICs can assemble correctly at the sperm nuclei, but then are not able to migrate properly to individualize sperm tails.

Myosin VI (myo VI) is known to stabilize the actin cones during spermatogenesis and the activity of this motor is essential during individualization (Hicks et al., 1999; Noguchi et al., 2006). Since defects in the migration of the actin cones were noticed in *Bug22* sperm, we investigated whether myo VI recruitment or localisation was perturbed in mutant sperm tails. Just like in WT, myo VI was correctly recruited to the actin cones at IC assembly and relocalised to the front of the cones during IC migration (Fig. 5A and insets) in *Bug22* flagella. Taken together, characterization of *Bug22* cysts undergoing spermatid differentiation both by TEM and immunofluorescence showed that Bug22 is essential during this step of sperm maturation.

### The incorporation of the post-translational modification, polyglycylation is defective in *Bug22* mutant flagella

The function of Bug22 in IC movement could be linked to its localisation to sperm tails, likely at the level of the axonemal MTs. A clear evidence for the ability of this protein to bind the MT cytoskeleton has been provided in a proteomic study of *C. reinhardtii* flagella (Pazour et al., 2005), in which Bug22 ortholog was found enriched mainly in the fraction of proteins that were more strongly associated with the axoneme. Although not much is known about the MT properties that allow the movement of actin cones along the axoneme, tubulin polyglycylation, a post-translational modification (PTM) (Redeker et al., 1994) mostly present in ciliary MTs, was suggested to play a direct role in IC migration and in *Drosophila* sperm individualization (Rogowski et al., 2009). The same study also showed that reduced levels of glycylation cause the formation of a structurally normal but unstable sperm axoneme, similarly to what we saw in *Bug22* flies.

*Drosophila* sperm tails are known to contain high levels of polymodified MTs, namely of polyglutamylation and polyglycylation (Bressac et al., 1995; Hoyle et al., 2008; Kavlie et al., 2010; Rogowski et al., 2009), particularly the accessory and central pair of MTs in the sperm axoneme (Hoyle et al., 2008). Therefore, we decided to determine whether these modifications were correctly incorporated in *Bug22* axonemes. In western blot and immunostaining procedures, using antibodies that recognize long chains of glutamate residues (polyE) to detect polyglutamylation (Eddé et al., 1990; Janke and Bulinski, 2011), we obtained inconclusive results that were highly variable. On the other hand, when we analysed polyglycylation, using polyG antibodies that recognize long glycine chains (3 and more residues) (Eddé et al., 1990; Janke and Bulinski, 2011; Redeker et al., 1994), we found a strong reduction in the mutant (Fig. 5B,C; supplementary material Fig. S2A). Since in extracts that combined testes from different males, a large variation in signal was noticed (data not shown), we analysed four independent experiments in which samples of single testes from WT and *Bug22* flies were compared (Fig. 5B,C; see Materials and Methods). This procedure revealed a significant reduction of polyglycylation in the mutant. Importantly, this decrease was never accompanied by equivalent alterations in the total levels of tubulin, which appeared similar to WT in *Bug22* testes (Fig. 5D). Interestingly, the difference in PTM levels was not only restricted to α- and β-tubulins, as the polyglycylation levels of other yet unidentified proteins of higher molecular weight were also reduced in the mutant (Fig. 3B, arrow).

We then analysed the distribution of polyglycylation in WT and *Bug22* cysts. Surprisingly, we noticed the occurrence of highly complex patterns of tubulin polyglycylation in WT testes reflecting a developmentally regulated process. A detailed analysis of the precise timely incorporation of this tubulin modification during *Drosophila* sperm maturation has never been reported. Therefore, we decided to characterise polyglycylation (using polyG antibodies) in semi-squashed preparations (see Materials and Methods). We focused our analysis in the period of spermatogenesis that ranged from the initial phases of sperm differentiation, when sperm tails start to elongate, until the final stages, where fully mature individual sperm cells coil and separate from each other, in order to be mobilised into the seminal vesicle (Fabrizio et al., 1998; Tokuyasu et al., 1972a; Tokuyasu et al., 1972b) (Fig. 5E).

After having subdivided the spermatogenesis period in four different stages following to the distinct nuclear morphologies progressively acquired by the maturing spermatids (see Materials and Methods for a detailed analysis of the sub phases), analysis of WT cysts revealed that, from the beginning of elongation until the formation of individual coiled sperm cells, polyglycylation appeared according to a defined order. In pre-elongation stages, polyglycylation was relatively low with a clear signal surrounding each nuclei (Fig. 5F,G) both in WT and mutant cysts. In the canoe stage, however, polyglycylation levels were increased in the mutant, suggesting premature polyglycylation of *Bug22* sperm tails. At later stages, polyG signals never reached WT levels (Fig. 5F,G). In *Drosophila*, two enzymes, TTLL3A and TTLL3B, are able to initiate and elongate glycine chains in opposition to mammals, where separate enzymes specifically catalyze each step: initiation (TTLL3/8) or elongation (TTLL10) (Rogowski et al., 2009). Previous studies suggested that polyglycylation occurs during IC passage and that the enzymes responsible for this modification could be transported by these complexes (Bressac et al., 1995; Rogowski et al., 2009). In light of this hypothesis, it was possible to conceive that some of the defects in polyglycylation, observed in *Bug22* mutants could result from defects in IC migration. To investigate this question we analysed whole-mount preparations of WT testes co-stained for F-actin and PolyG. In sperm tails that contain ICs that were just starting to assemble near the sperm nuclei, polyglycylation was already detected along sperm tails (supplementary material Fig. S3). Furthermore, when we analysed sperm tails that contained migrating IC cones positioned away from the sperm nucleus, a strong and comparable polyG signal was noticed both in front and at the rear of the IC complex (supplementary material Fig. S3). Altogether, these results showed that sperm tail polyglycylation takes place earlier than initially proposed, either before or during IC recruitment to the sperm head but, importantly, before IC passage. These observations lead us to conclude that polyglycylation is incorporated independently of IC passage. Interestingly, this first description of *in vivo* PTM regulation during *Drosophila* spermatogenesis is in agreement with *in vitro* data describing the appearance of tubulin polyglycylation independently of IC passage (Hoyle et al., 2008).

**Fig. 6.**
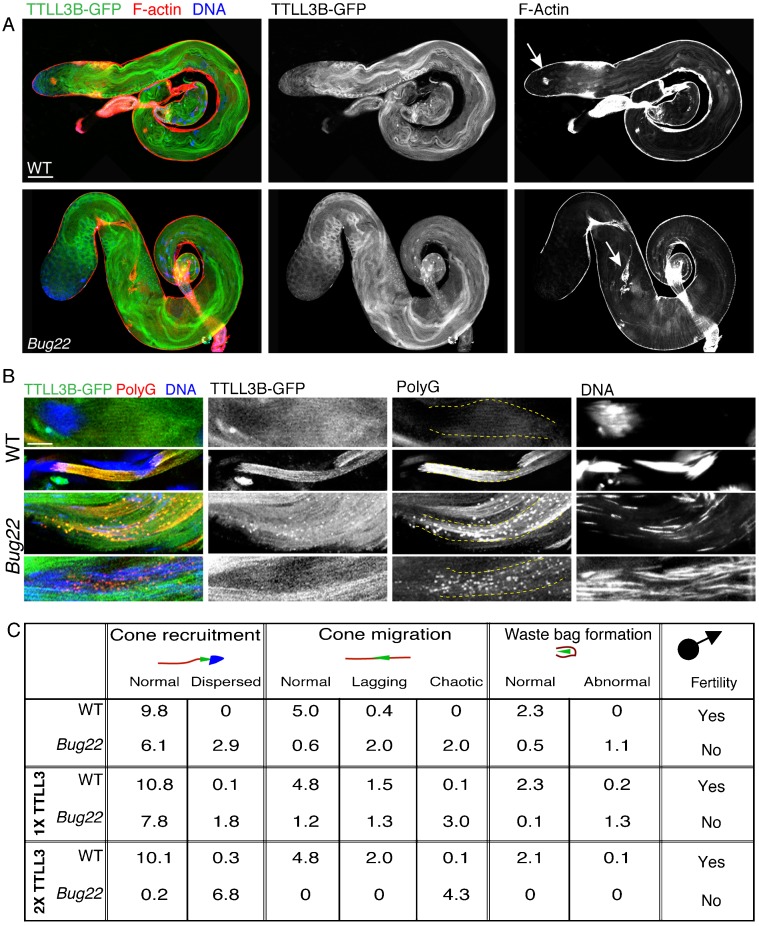
TTLL3B-GFP overexpression enhances the defects of *Bug22* testes. (A) Whole mount staining of TTLL3B-GFP expression in WT (upper image) and *Bug22* (bottom image) testes stained for F-actin (red) and DNA (blue). Scale bar: 100 µm. (B) Images from TTLL3B-GFP, WT (upper two panels) and TTLL3B-GFP,*Bug22* (bottom two panels) elongating and individualizing cysts, stained for polyG (shown in red) and DNA (shown in blue). The ectopic expression of TTLL3B-GFP in the *Bug22* background resulted in the appearance of polyglycylated aggregates that accumulate along the cyst during the canoe stages. Sperm nuclei appeared dispersed and the whole cyst appeared very disorganised. Scale bar: 5 µm. (C) Quantification of IC recruitment, migration and waste bag formation (using phalloidin and caspase-3 staining) in WT and *Bug22* testes expressing either one or two copies of TTLL3B-GFP or without expression of TTLL3B-GFP. Numbers refer to average amount of ICs of each category per testis.

Our results suggested that Bug22 plays an important function in maintaining polyglycylation levels in *Drosophila* sperm tails. To ascertain if the defects observed in polyglycylation could be rescued by overexpression of glycylating enzymes, we overexpressed a GFP fusion of TTLL3B using a ubiquitous promoter that induces moderate overexpression (Basto et al., 2008; Peel et al., 2007). Overexpression of TTLL3B-GFP in WT flies did not have any deleterious effect. Flies eclosed normally, displayed normal climbing and flying activity and both female and males were fertile. In contrast, the overexpression of TTLL3B-GFP in *Bug22* had a deleterious effect. These flies were delayed during development and most flies died at late pupal stages or just after eclosion. These results suggested that the overexpression of TTLL3B could not rescue the individualization phenotype of the mutant. Examination of TTLL3B-GFP,*Bug22* testes showed an aggravation of the spermatogenesis phenotype when compared to *Bug22* testes (Fig. 6C). IC recruitment and migration were severely perturbed and nuclei appeared dispersed along the sperm tails. In the few partially intact cysts in the canoe/needle stages that we were able to analyse (Fig. 6B), high-intensity polyG aggregates were noticed accumulating in vesicle-like structures that also contained TTLL3B-GFP, suggesting ectopic, exaggerated and premature enzyme activity. Importantly, the later stages of sperm maturation, such as post-elongation, were only rarely seen in TTLL3B-GFP,*Bug22* testes.

Furthermore, we noticed a dose-dependent effect of TTLL3B-GFP transgenes when expressed in *Bug22* mutant background (Fig. 6C). When two copies were present, IC recruitment to the sperm nuclei and migration (analysed by the morphology and position of the ICs) were severely impaired and we never observed the formation of waste bags when compared to testes that only contained one TTLL3B-GFP copy (Fig. 6C). These results suggest that the overexpression of TTLL3B in the absence of Bug22 results in premature and (severely increased) polyglycylation during the canoe stages, which causes extreme defects in spermatogenesis.

### Regulation of ciliary tubulin modifications by Bug22 is conserved in vertebrate cells

**Fig. 7.**
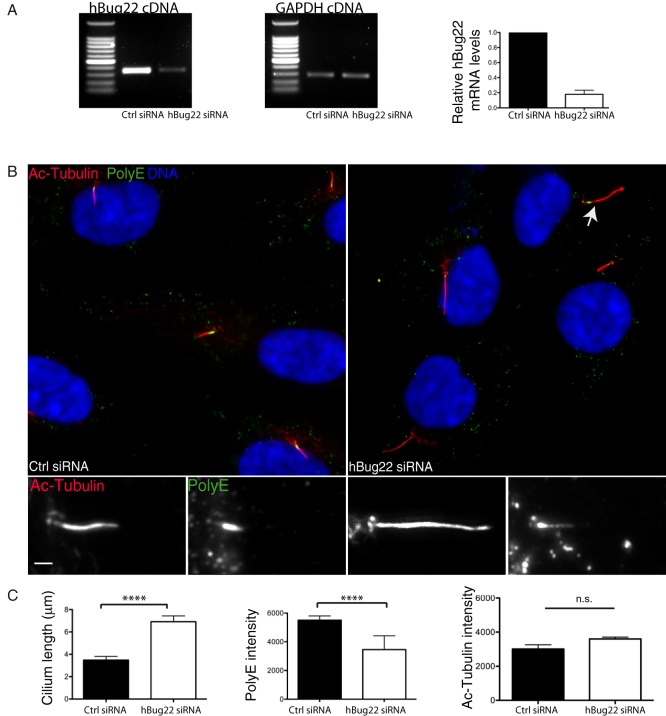
Depletion of the human Bug22 ortholog increases the length of primary cilia. (A) Estimation of hBug22 depletion by RT-PCR analysis in RPE1 cells after 72 h treatment of hBug22 siRNAs and negative control (ctrl). *GAPDH* was used as loading control. The graph bars show average ± SEM from three independent experiments. (B) Immunostaining pictures of hBug22 siRNA treated RPE1 cells stained with Ac-Tubulin (shown in red) and polyglutamylated tubulin (polyE) (shown in green) and for DNA (show in blue). Insets represent three-fold enlarged regions of the main image. Scale bars: 3 µm. (C) Analysis of cilium length and fluorescence intensity of ciliary polyglutamylated tubulin (revealed by polyE antibodies) and Ac-Tubulin in control and hBug22 siRNA treated cells. Bars show average ± SEM from two independent experiments (*n*≥35 per experiment). The length of cilia in hBug22 depleted cells were significantly higher as compared to control cells (unpaired Student's t-tests, two-tailed, *P*<0.0001). A statistically significant reduction in polyE levels was also found in hBug22-depleted cells (unpaired Student's t-tests, two-tailed, *P*<0.0001). In contrast, Ac-Tubulin levels were not significantly changed in hBug22 depleted cells (unpaired Student's t-tests, two-tailed, *P*>0.1).

Given the high degree of conservation between the human and fly Bug22 orthologs (92%) and the ciliary localisation of hBug22 in hTERT-RPE1 cells (Fig. 1D and Ishikawa et al., 2012), we decided to test whether primary cilium tubulin PTMs were also dependent on Bug22. PTMs are known to be essential for normal cilia function and architecture. Therefore, we hypothesized that if a function for Bug22 in regulating PTMs was conserved in vertebrates, cilia defects should be seen in the absence of Bug22. To start this analysis we characterised PTMs in primary cilia of RPE1 cells. Using polyG, polyE and acetylated tubulin antibodies we observed that the last two antibodies recognise centrioles and primary cilia, suggesting that in RPE1 cells these structures are polyglutamylated and acetylated but not polyglycylated. Knockdown of hBug22 using small interfering RNAs (siRNAs) resulted in a ∼80% decrease in Bug22 levels as determined by RT-PCR (Fig. 7A).

Analysis of hBug22-depleted cells revealed the presence of a large proportion of cells that grew longer cilia (Fig. 7B,C). Furthermore, these cilia frequently displayed a curved morphology that was never seen in control cells (Fig. 7B, arrow). We did not find differences in the levels of acetylated tubulin in hBug22-depleted cells (Fig. 7C). In contrast, the levels of polyE were significantly decreased (Fig. 7C) in the longer cilia. Importantly, we were able to rescue ciliary size defects in hBug22-depleted cells by expressing an siRNA-resistant GFP-hBug22 fusion protein (supplementary material Fig. S4), showing the specificity of the cilia size and morphology phenotypes. We conclude that in both *Drosophila* and human cells, Bug22 plays an essential role in maintaining cilia morphology, which might depend on the correct incorporation of tubulin PTMs.

## Discussion

Here we have investigated the role of Bug22 proteins in *Drosophila* and RPE1 ciliogenesis. In both experimental models, we demonstrated a requirement for Bug22 in maintaining ciliary morphology, as well as defects in the levels of tubulin PTMs. We show that in flies, Bug22 modulates the timely incorporation of polyglycylation during spermatogenesis.

### Analysis of *Bug22* mutants

*Bug22* mutants did not show defects during development. At birth, however, *Bug22* flies could be subdivided into two main classes. An Unc-type class, where mutant flies, similarly to other cilia mutants, were uncoordinated and presented defects in locomotion, gravitaxis and were unable to feed. These flies died within a few days after eclosion, probably do to dehydration, similarly to other cilia mutants (Baker et al., 2004; Martinez-Campos et al., 2004; Basto et al., 2006). Unexpectedly, the other class of *Bug22* mutants presented an even more severe phenotype. They were unable to inflate their wings, presented defects in cuticle deposition and remained paralyzed. They invariably died just a few hours after eclosion. This type of defects has not been reported in centrosome or cilia mutants and we think they result from a yet uncharacterised function of Bug22. In flies, two types of non-visual sensory organs, Type-I (also known as sensilla), that harbor ciliated neurons and Type II, which consist of single, non-ciliated multi-dendritic neurons can be found (Avidor-Reiss et al., 2004; Gogendeau and Basto, 2010; Kernan et al., 1994; Kernan and Zuker, 1995). Since all *Bug22* defects were rescued when we specifically overexpress Bug22 with pan-neuronal Gal4 drivers (Fig. 2C), but not with the JO15Gal4 (expressed in the Johnston organ, which contains only ciliated neurons), we propose that this protein probably plays essential functions in both ciliated and non-ciliated neurons.

### *Bug22* mutants present overly long centrioles and hBug22 depletion causes lengthening of primary cilia

During the course of this study we found that *Bug22* centrioles and basal bodies were longer than the equivalent WT organelles (Fig. 3D,E). In addition, during meiotic stages, *Bug22* centrioles lose their typical V-shape. Importantly, defects in centriole assembly were not found in any other cell type in B*ug22* mutants (data not shown), not even in the mitotic stages that precede the formation of the large primary spermatocyte centrioles. The abnormal lengthening probably reflects the unusual property of these centrioles, which is the nucleation of a primary cilium at their most distal end (Tates, 1971; Varmark et al., 2007; Carvalho-Santos et al., 2012; Riparbelli et al., 2012). Mutations in centriole components are known to perturb meiotic divisions or to cause fragmentation of primary spermatocyte centrioles, which leads to multipolar spindle formation (Bettencourt-Dias et al., 2005; Delgehyr et al., 2012; Martinez-Campos et al., 2004; Riparbelli and Callaini, 2011; Rodrigues-Martins et al., 2007). However, this was not the case in *Bug22* mutants, as we have never observed defects in spindle assembly or chromosome segregation (data not shown) consistent with the lack of abnormalities at the onion stage (Fig. 3C).

Depletion of hBug22 in RPE1 cells resulted in the formation of an elongated primary cilium. In control cells, a strong polyglutamylation signal was detected at the proximal end just above the transition zone. In hBug22-depleted cells, a significant decrease in polyglutamylation was noticed, while another modification, acetylation, was unchanged. Furthermore, some of these cilia lost their normal morphology and appeared curved, similarly to *Bug22* depleted *Paramecium* cells (Laligné et al., 2010). Together, these results show that Bug22 is not essential to build the axoneme but it is essential to determine its final size and the overall morphology of the cilium. Possibly, axonemal length control depends on tight regulation of tubulin PTMs. Few studies reported so far on lengthening of primary cilia or even motile cilia. In *Chlamydomonas*, mutants with abnormal flagella lengthening display defects upon flagella regeneration (Tam et al., 2007), whereas in vertebrates, both Broad-minded and Nde1 knockdowns, which also cause abnormal lengthening of primary cilia, impact on cell cycle progression (Kim et al., 2011) and sonic hedgehog signaling (Ko et al., 2010). It is thus important to control cilia size and this study has identified Bug22 as an essential player in the determination of cilia size.

### Bug22 plays a role in sperm individualization

All *Bug22* males produce immotile sperm that contained correctly assembled axonemes most of the time (Fig. 4B–D). Defects in sperm individualization were noticed, which did not result from defects in the initial recruitment of ICs. Instead, defects in IC migration were frequent, and the waste bags that normally form at the end of individualization, were either small or absent (Fig. 5A). Together these results suggest that the initial recruitment of the individualization machinery takes place in *Bug22*, although the synchronous migration is compromised.

When we analysed tubulin polyglycylation in *Bug22* we found two types of defects. First, polyglycylation was deposited prematurely during the canoe stages, while in WT sperm tails this modification was noticed mainly at the needle stages (Fig. 5F,G). Then, at late stages, during the needle and post-elongation periods, polyglycylation levels did not reach high levels in the mutant (Fig. 5F,G). Thus, it is possible that the initial addition of the modification can take place in *Bug22* axonemes even if prematurely, but the second polyglycylation wave (or the elongation of glycine chains) during the post-individualization stages fails.

The observations that overexpressing TTLL3B-GFP results in the formation of polyG aggregates along the sperm tail only in the mutant support a role for Bug22 in exerting a buffering effect in order to control (or timely modulate) the activity of TTLL3B in the *Drosophila* sperm tail. It is possible that Bug22 functions as a filter that occupies the axoneme surface to work as a steric hindrance that limits the access of active enzyme to MTs, functioning as a fine-tuning mechanism for TTLL3B activity. Although other approaches are required to understand the biochemical functions of Bug22 and its interaction with tubulin-modifying enzymes, a simple interpretation of our results is that Bug22 plays a function in the elongation of polyglycyl chains. In WT sperm, polyglycylation starts being detected at needle stages increasing slightly at post-elongation stages. In the absence of Bug22, initiation of glycylation and initial extension of the glycyl chains (at levels that can be detected by polyG) takes place during canoe stages, prematurely to WT, suggesting a function in controlling the timely incorporation of the modification. It is worth noting that since polyglycylation of other proteins in testes extracts is also affected in *Bug2*2 when TTLL3B-GFP is overexpressed, it is possible that the defects observed in IC recruitment at the sperm nucleus (Fig. 6B) might also depend on these substrates.

## Conclusions

Our work shows that Bug22 influences the size of organelles that contain an axoneme such as centrioles and basal bodies (the unusual primary cilium present in fly centrioles and basal bodies), both in the male germline in *Drosophila* as well as the primary cilium of RPE1 cells. Very likely, Bug22 also contributes to the morphology of other MT structures that are not organised in axonemes, as expression of Bug22 in neurons rescued the severe phenotypes associated with the *Bug22* mutation. MTs in neurons are highly modified and stabilized (Janke and Bulinski, 2011) and so, Bug22 likely plays a function in these cells.

Bug22 is remarkably conserved across evolution and it is even present in non-ciliated genomes as seed plant genomes (Hodges et al., 2011; Laligné et al., 2010). It will be interesting in the future to understand whether Bug22 also influences tubulin PTMs in cell types or organisms that do not contain cilia.

## Materials and Methods

### Fly stocks

All flies were maintained and handled according to standard *Drosophila* culture techniques. Stocks used in this study: *P[Ubq::RFP-PACT]/CyO* (Martinez-Campos et al., 2004), *P[Ubq::GFP-Bug22]*, *P[UAST::GFP-Bug22]*, *P[Ubq::TTLL3B-GFP]*, *P[Bug22^KO^]/TM3,Sb*, *Bug22*/*CyO*, *Bug22*/*CyO,P{Ubi-GFP.S65T}PAD1* (this study), y^1^w*/Y, hs-hid; P[70FLP]23 P[70I-SceI]4A*/TM3 Sb hs-hid* (Huang et al., 2008), w*; Pin^1^/CyO;P{GawB}^221w^^-^, w*;P[GAL4-elav.L]3,w*;P[J21.17-GAL4]JO15/TM3,Sb^1^,*w^1118^;P[GawB]l(2)DJ684^DJ684^/CyO*, y^1^w*;P[GAL4-Mef2.R]3, w*; P[GawB]how^24B^, from the Bloomington Stock Center. *w^1118^* flies were used as WT controls.

### Generation of *Bug22* mutant by homologous recombination

For generation of the *Bug22* knockout allele, we used the strategy described in Huang et al. (Huang et al., 2008). Briefly, a transgenic fly harboring a “donor DNA” construct to be used for gene targeting was made. To this end, a fragment of 3155 bp of genomic sequence with its 3′ limit at the end of *Bug22* gene (position Ch2L: 10429989) was PCR-amplified from *w^1118^* genomic DNA with primers 5′-ATACGGTACCCCGCGGATCATGTGGCGGCACTTATC-3′ and 5′-ATACCTGCAGCATATGTGCACAAGACTGCCATCCAGCACTCATTC-3′ using Phusion DNA Polymerase (no. F-530, Themoscientific), subsequently sequenced and cloned into the 5′ multiple cloning site of the pRK2 plasmid (Huang et al., 2008), using SacII and NdeI sites. A second insert was cloned into the 3′ cloning site of the same vector using BglII and PstI sites. This 3035 bp fragment, contains the 5′ limit flanking the *Bug22* ATG starting codon (position Ch. 2L: 10430817), was amplified with primers 5′-ATACGGTACCAGATCTCGAAACTCGTACAAACTACG-3′ and 5′-ATACCTGCAGTGGGTCCTAGATCCAGTTAATG-3′. This final pRK2-P*{Bug22^KO^}* transformation vector, was used for transgenesis by Bestgene Inc., USA.

A transgenic line carrying the transgene on chromosome III was used for homologous recombination. Putative *Bug22* mutant lines, homozygous for the “donor”-DNA construct at chromosome II, were screened for by genomic DNA PCR using primers 1+2 (5′-GTTCGAGCACACCATTCTGA-3′, 5′-TGATAGGAATCCCGATTGGA-3′) and 3+4 (5′-ACTTTCCAATCGGGATTCCT-3′, 5′-TTACGGCCAACCTTAACTGG-3′). More details are provided in supplementary material Fig. S1.

### Generation of Bug22 transgenes

Constructs used to generate transgenic lines expressing fluorescently tagged Bug22 were produced by cloning the *CG5343* coding sequence, amplified by PCR from *w^1118^* genomic DNA using Phusion DNA Polymerase (no. F-530, Themoscientific), with the following primers: 5′-GGGGACAAGTTTGTACAAAAAAGCAGGCTCAATGTTCAAAAACACTTTCCAATCG-3′ and 5′-GGGGACCACTTTGTACAAGAAAGCTGGGTCGCTACAAATCGCATTGG-3′. After sequencing, the PCR fragments were cloned into Gateway vectors pUbq-GFPNT Gateway vector (Basto et al., 2006) for generating pUbq-GFPBug22 lines and pTWG (DGRC) for generating pUAST-GFPBug22 lines. Transgenic lines expressing TTLL3B-GFP were made from a construct carrying artificially synthesized TTLL3B cDNA (Genscript USA Inc.) that was cloned into the pUbq-GFPCT Gateway vector (Peel et al., 2007). The final constructs were sent for transgenesis to Bestgene Inc., USA.

### Fertility and Unc-phenotype tests

At least 15 vials containing single males of a given genotype were allowed to mate with two/three *w^1118^* females. Vials were kept at 25°C. In the case of female fertility tests, the reciprocal cross was made with *w^1118^* males. Hatching of embryos as first instar larvae was followed for 72 h (normal hatching time at 25°C is 24 h). The phenotype classes were defined in the following way: wings that were closed like WT ones were considered “normal”, wings that appeared permanently opened were considered “upheld” and when these were folded, they were termed “non-inflated”. As for the climbing activity phenotypic classification, flies were termed normal when were undistinguishable from WT, “lack of climbing capacity” when flies stayed permanently at the bottom of the culture vials, “lack of equilibrium when standing” or “slowness” if they displayed each of these intermediate movement coordination phenotypes.

### Electron microscopy

Testes from male pupae (at about 80 h of pupariation) and from adult males (2 days after eclosion) were dissected in PBS and were fixed in chilled glutaraldehyde (2% in 0.1 M phosphate buffer, pH 7.4) overnight. After a 30 min wash, samples were post-fixed in 1% OsO_4_, dehydrated in graded concentrations of ethanol and subsequently embedded in Epon 812 resin (no. T024, TAAB). Polymerisation at 60°C for 48 h followed. Ultrathin sections of the specimens were collected on copper grids, and stained with uranyl acetate and lead citrate. Sample analysis was done using a Philips CM120 electron microscope (FEI, Eindhoven, Netherlands). Image acquisition with a KeenView camera (SIS, Munich, Germany) and measurements were made with the iTEM software (Olympus France SA, Rungis, France).

### Immunoblotting

Testes extracts were prepared either by dissecting 16 testes into 50 µl of cold PBS (with 1 mM PMSF and protease inhibitor cocktail from Sigma), to which 50 µl of 2× Laemmli buffer was added, or by dissecting one single testis and transferring it directly to 30 µl 2× Laemmli buffer . Samples were denatured by boiling for 5–10 min. Equal volumes of sample from different genotypes were loaded into 10% precast NuPAGE Bis-Tris Gels (Invitrogen). Samples were blotted onto a nitrocellulose membrane (Whatman) and probed with antibodies used as follows: GTL3 (anti-Bug22) at 1/2500 (no. ab33872, Abcam), DM1A (anti-α-tubulin) at 1/5000 (Sigma), polyE (anti-polyglutamylation recognizing long chains of at least three residues or more) at 1/9000 (C. Janke, Institut Curie), polyG (anti-polyglycylation) at 1/9000 (M. Gorowsky, University of Rochester, NY), GT335 at (anti-polyglutamylation; recognizes both short and long chains) at 1/6000 (Enzo life Sciences-800855C100) and Rabbit/Mouse anti-HRP at 1/5000 (Jackson ImmunoResearch Laboratories, Inc.). Detection was made with Amersham ECL Plus Western Blotting Detection Reagents (GE Healthcare) or SuperSignal West Pico Chemiluminescent Substrate (Pierce). Quantification of the intensity of bands on gels was done using the “Gel analysis” tool from ImageJ, according to the instructions present in http://lukemiller.org/index.php/2010/11/analyzing-gels-and-western-blots-with-image-j. Mean intensity values for each sample at the level of the band corresponding to polyglycylated tubulin, and for the corresponding representative band from the Coomassie-stained gel, were obtained. These two values were used to determine the amount of modified tubulin per amount of protein loaded followed by normalization.

### Immunohistochemistry and microscopy

Preparation of fly antenna from *P[Ubq::GFP-Bug22]* pupae (at about 50 h post-pupariation) was made as described (Martinez-Campos et al., 2004). Samples were additionally stained for DNA with Hoechst 33258 (0.5 µg/ml in PBS, Invitrogen), for 15 min and mounted on slides with 12 mm-round coverslips.

For semi-squashed testes preparations, testes and attached seminal vesicles were dissected from pupal or 1–2-day-old flies, after which they had their sheath opened through a strong pull made with the dissecting forceps. Samples were allowed to settle for a few seconds and were then mounted in PBS between a coverslip and a microscope slide. Removal of excess buffer with tissue paper promoted enough squashing, which allowed for detailed visualization of cells and cysts at the different steps of spermatogenesis and also for checking for motility of sperm/spermatid tails. Processing for immunofluorescence was carried out using two different methods. In the cases where GFP/RFP fluorescence had to be kept, testes were immediately fixed as described for brain squashes (Sabino et al., 2011) except that the acetic acid step was omitted and the tissue gently squashed. In the other cases, testes were prepared after dissection as described (Pisano et al., 1993), with minor modifications: the squashing step was done on slides coated with 5% poly-lysine solution, and the blocking solution was 3% BSA in PBT (PBS + 0.1% Triton X-100). Incubations with primary antibodies were done overnight at 4°C and with secondary antibodies for 2 h at room temperature (RT) in moist chambers, and Hoechst staining was done for 5 min in PBT. Finally, slides were allowed to dry and mounted in 10 µl medium (1.25% (m/m) N-propyl gallate, 30 ml glycerol, 10 ml H_2_O).

For whole mount testes preparations, testes, and attached seminal vesicles, from 1–2-day-old flies were dissected in PBS and processed as described (Texada et al., 2008). After secondary antibody washes, testes were incubated with Hoechst/PBS solution for 10 min. Samples were washed in PBS and mounted with 8 µl of mounting medium with 12 mm-round coverslips.

RPE1 cells that had been cultured on acid-treated glass coverslips were washed once with PBS and fixed, either with methanol kept at −20°C for 6 min, or with RT 4% paraformaldehyde for 10 min. After 3 washes of 5 min each with PBS, cells were permeabilised with PBT and blocked with PBT + 3% BSA solution. Incubation with primary antibody mixtures diluted in the blocking solution was carried out for two hours at RT. Cells were then washed 3 times with PBT, incubated with secondary antibody dilutions in blocking solution for 1 h at RT, and finally, incubated with Hoechst/PBT solution for 5 min. After a PBS wash, slides were dried and samples mounted with 8 µl mounting medium.

Antibody incubations were made at the following dilutions: 21A6 (anti-Eys) at 1/50 (Developmental Studies Hybridoma Bank), DM1A (anti-α-tubulin) at 1/1000 (Sigma), anti-Cleaved Caspase-3 at 1/200 (no. 9664, Cell Signaling Technologies), anti-*Drosophila* myosin VI at 1/100 (3C7, a courtesy of K. G. Miller, Washington University), GTU88 (anti-γ-tubulin) at 1/500 (Sigma), polyE at 1/1000, 6-11B-1 (anti-acetylated tubulin) at 1/1000 (Sigma). Secondary antibodies used were Alexa Fluor 488 anti-rabbit, Alexa Fluor Cy3-conjugated anti-mouse, Alexa Fluor 568 anti-mouse and Alexa Fluor 568 anti-rabbit, Cy5-conjugated anti-rabbit all at 1/500 (Molecular Probes). Alexa Fluor 488 phalloidin at 1/100 (Invitrogen) stained F-actin and Hoechst 33258 stained DNA.

Examination of testes by phase contrast microscopy was performed in an Eclipse Ti Inverted Microscope (Nikon), equipped with a piezo-electric driver mounted underneath the objective and a CoolSNAP HQ2 camera (Photometrics) and a 10× or 40× phase-contrast objective. The microscope was controlled by the Metamorph imaging software (Molecular Devices).

Squashed testes and RPE1 cells were analysed in an epifluorescence microscopy unit, composed of an Eclipse 90i Upright Microscope (Nikon), equipped with a 100× oil immersion objective, a piezo-electric driver mounted underneath the objective and a CoolSNAP HQ2 camera (Photometrics). The microscope was controlled through the Metamorph imaging software (Molecular Devices). Acquisitions of Z-series with 0.2 µm increments were performed.

Fly antenna and whole mount testes were imaged with a confocal system mounted on an Eclipse Ti inverted microscope (Nikon), and equipped with a MCL Piezo stage and 20×, 40× and 60× lenses. This microscope was controlled by the NIS-Elements software (Nikon). While analysing tubulin PTMs, the same laser and Z-stack acquisition settings were used for all specimens of each experiment.

Image files were first processed with ImageJ software, which allowed building a maximum intensity projected image from selected Z-series slices acquired. Further combination of channels into RGB colour images and adjustment of black and white input levels for each channel, in order to remove background signal and improve image visualization, were made in Adobe Photoshop CS4 software (Adobe). In all cases, control and experimental images were treated in the same way.

### Cell culture

hTERT RPE-1 (RPE1) cells (a kind gift from Michel Bornens, Institut Curie) were cultured in DMEM/F-12 medium (Invitrogen), supplemented with penicillin/streptomycin (Invitrogen) and 10% fetal calf serum (Invitrogen), at 37°C and 5% CO_2_.

### DNA constructs, siRNAs and transfections

To produce a pEGFP-Bug22 construct, the full-length human Bug22 cDNA (kind gift from Jean Cohen, CGM, France) was amplified by PCR using Phusion DNA Polymerase (Finnzymes) and primers 5′-ATACCTCGAGCTATGTTCAAAAACACGTTCCAGAGC-3′ and 5′-ATACCCGCGGTTGCTTTGCCTTGTTCTGAAC-3′, sequenced and cloned in frame with EGFP coding sequence into the XhoI and SacII sites of pEGFP-C1 vector (Clontech). Equivalent cloning procedures were followed to produce the pEFP-Bug22RR, except that the Bug22 template used for cloning, which contained silent mutations rendering it resistant to siRNA knockdown, was created by DNA synthesis (Genscript USA Inc.). Plasmid DNA transfections were done as follows: one day before transfection, RPE1 cells were seeded at a density of 0.8×10^5^ cells/well, in 24-well plates, containing acid treated, sterile glass coverslips in each well. Transfection of 0.2 µg pEGP-hBug22 or pEGFP-C1 plasmids was then done using lipofectamine 2000 (Invitrogen), according to the manufacturer's instructions. Complexes were removed after 6 hours and cell medium was replaced by DMEM/F-12 medium with a low serum dosage (0.5%), and cells were incubated for another 24 h before being processed for immunofluorescence.

Pre-designed siRNA oligonucleotides from Qiagen were used in our assays: SI04344613 (TCGTCGCTTTCGGGCAAGTAA, FlexiTube siRNA) and SI00432383 (CAGGTACTAGATGACAAGAAT, FlexiTube siRNA) targeted Bug22, whereas SI03650318 (AllStars Negative Control, FlexiTube siRNA) was used as a control for transfection. For transfection, RPE1 cells were seeded at a density of 0.5×10^5^ cells/well in 24-well plates, containing acid treated, sterile glass coverslips. The following day, transfections were carried out with Lipofectamine™ RNAiMAX (Invitrogen), according to the manufacturer's instructions, using siRNAs at a final concentration of 10 nM in low serum (0.5%) DMEM/F-12 medium. Cells were incubated for 72 h and then processed for immunofluorescence and gene knockdown analysis by RT-PCR.

Cotransfection of plasmid DNA and siRNAs was done as for plasmid DNA alone. After 24 h of treatment, the culture medium was replaced by DMEM/F-12 medium supplemented with 0.5% FBS, and cells were kept for additional 48 h until being processed for immunofluorescence and gene knockdown analysis.

### Semi quantitative RT-PCR

Total RNA from RPE1 cells was extracted with the RNeasy Minikit (Qiagen), according to the manufacturer's instructions. Cells from 3 wells of 24-well plates were used for each RNA sample preparation. RNA was resuspended in 30 ml of nuclease-free water and its concentration measured in NanoVue Plus Spectrophotometer (GE Healthcare). cDNA synthesis was done using the High-Capacity cDNA Reverse Transcription kit (Applied Biosystems), according to manufacturer's instructions, except for the fact that oligodT(20) primers (Sigma) were used instead of the random primers provided. cDNA was kept at −20°C or used for PCR with GoTaq DNA polymerase (Promega) in 25 ml mixtures. Preliminary PCR amplification was carried out, using a fixed amount of cDNA template and a different number of PCR cycles, in order to determine the linear range of amplification of each product. For the *hBug22* amplification, 50 ng of cDNA sample were used with primers TGGCCACATCAAAAGAATCA and TCGATGTAATTGGTGCCGTA at 0.40 mM each, for 26 cycles of amplification. In turn, for the amplification of *GAPDH*, 25 ng of the cDNA with primers CTGCACCACCAACTGCTTAG and AGGTCCACCACTGACACGTT at 0.40 µM each were used for 20 cycles of amplification. Amplified DNA fragments were separated and visualized in 0.8% agarose gels in TAE buffer.

Quantification of the intensity of bands on gels was done using the “Gel analysis” tool from ImageJ, according to the instructions present in the following website: http://lukemiller.org/index.php/2010/11/analyzing-gels-and-western-blots-with-image-j.

### Quantifications of centriole/cilia lengths

Measurements of centriole and cilia lengths were made in ImageJ software, using the macro 3D-Distance Tool. After setting the voxel dimensions of each Z-series acquisition, the limits of fluorescence signal of the centriole marker considered for the measurement were chosen manually. We used RFP-PACT and Asterless to label centrioles and basal bodies in both meiotic spermatocytes or early spermatids and acetylated tubulin to label primary cilia in RPE1 cells.

### Fluorescence intensity measurements

Mean fluorescence intensities for stained PTM tubulins were measured using the measure tool of the ImageJ software. For measurement in spermatid tails, a representative area covering tails from one cyst was selected. The threshold for the subarea corresponding to tubulin signal (filamentous) and to the background signal were then defined manually using the threshold dialog window and the final mean fluorescence intensity determined by subtracting from the thereby assigned mean signal intensity the corresponding mean background intensity. This procedure was partially automatised using a macro created by P. Gilloteaux (Institut Curie). Each dot in Fig. 5G corresponds to the mean intensity of a given cyst at a particular stage of development. For characterization of PTMs in the sperm testes, cysts were classified in four consecutive sub phases of sperm maturation, identifiable according to their nuclear shape (Lindsley, 1980; Rathke et al., 2010). We considered cysts to be in the pre-elongation cysts when they contained round nuclei, in the canoe stages when they were slightly elongated, in the needle stage when the nuclei were fully elongated and finally in the post-elongation stage, when nuclei were fully elongated but also packed closely together.

Primary cilium length was measured by drawing the segment tool in the acetylated tubulin (Ac-Tubulin) channel. The mean intensity for Ac tubulin was measured along the entire cilium length. PolyE signal is mainly proximal in RPE1 primary cilia. The mean intensity was measured along the proximal polyE positive signal. The intensity of three random circles surrounding the cilium in both channels was measured and their average used as background. The final mean fluorescence intensity was calculated by subtracting the mean background value from the mean signal value.

### Quantification of TTLL3B-GFP overexpression in testes

Testes were stained with DAPI to label nuclei, phalloidin to label actin, caspase-3 to label the sperm tails and waste bags. In the category “cone recruitment” we counted the number of cysts containing correctly assembled ICs (normal) or dispersed near the nucleus. In the category “cone migration”, we counted the number of cysts that presented either correctly migrating cones (normal), lagging (as shown in Fig. 5A-inset *Bug22*) or chaotic (as shown in Fig. 6A-*B**ug22*, arrow). Waste bag formation was ascertained by the presence of normal waste bags (as shown in Fig. 5A-WT) or abnormal (as shown in Fig. 5A-*Bug22*, arrowhead or in Fig. 6A, arrowhead).

## Supplementary Material

Supplementary Material

## References

[b1] Andersen J. S. Wilkinson C. J. Mayor T. Mortensen P. Nigg E. A. Mann M. (2003). Proteomic characterization of the human centrosome by protein correlation profiling. Nature 426, 570–574 10.1038/nature02166 14654843

[b2] Arama E. Agapite J. Steller H. (2003). Caspase activity and a specific cytochrome C are required for sperm differentiation in Drosophila. Dev. Cell 4, 687–697 10.1016/S1534-5807(03)00120-5 12737804

[b3] Avidor-Reiss T. Maer A. M. Koundakjian E. Polyanovsky A. Keil T. Subramaniam S. Zuker C. S. (2004). Decoding cilia function: defining specialized genes required for compartmentalized cilia biogenesis. Cell 117, 527–539 10.1016/S0092-8674(04)00412-X 15137945

[b4] Baker J. D. Adhikarakunnathu S. Kernan M. J. (2004). Mechanosensory-defective, male-sterile unc mutants identify a novel basal body protein required for ciliogenesis in Drosophila. Development 131, 3411–3422 10.1242/dev.01229 15226257

[b5] Basto R. Lau J. Vinogradova T. Gardiol A. Woods C. G. Khodjakov A. Raff J. W. (2006). Flies without centrioles. Cell 125, 1375–1386 10.1016/j.cell.2006.05.025 16814722

[b6] Basto R. Brunk K. Vinadogrova T. Peel N. Franz A. Khodjakov A. Raff J. W. (2008). Centrosome amplification can initiate tumorigenesis in flies. Cell 133, 1032–1042 10.1016/j.cell.2008.05.039 18555779 PMC2653712

[b7] Bettencourt-Dias M. Rodrigues-Martins A. Carpenter L. Riparbelli M. Lehmann L. Gatt M. K. Carmo N. Balloux F. Callaini G. Glover D. M. (2005). SAK/PLK4 is required for centriole duplication and flagella development. Curr. Biol. 15, 2199–2207 10.1016/j.cub.2005.11.042 16326102

[b8] Blachon S. Cai X. Roberts K. A. Yang K. Polyanovsky A. Church A. Avidor-Reiss T. (2009). A proximal centriole-like structure is present in Drosophila spermatids and can serve as a model to study centriole duplication. Genetics 182, 133–144 10.1534/genetics.109.101709 19293139 PMC2674812

[b9] Bressac C. Bré M. H. Darmanaden-Delorme J. Laurent M. Levilliers N. Fleury A. (1995). A massive new posttranslational modification occurs on axonemal tubulin at the final step of spermatogenesis in Drosophila. Eur. J. Cell Biol. 67, 346–355. 8521874

[b10] Broadhead R. Dawe H. R. Farr H. Griffiths S. Hart S. R. Portman N. Shaw M. K. Ginger M. L. Gaskell S. J. McKean P. G. (2006). Flagellar motility is required for the viability of the bloodstream trypanosome. Nature 440, 224–227 10.1038/nature04541 16525475

[b11] Carvalho-Santos Z. Machado P. Alvarez-Martins I. Gouveia S. M. Jana S. C. Duarte P. Amado T. Branco P. Freitas M. C. Silva S. T. (2012). BLD10/CEP135 is a microtubule-associated protein that controls the formation of the flagellum central microtubule pair. Dev. Cell 23, 412–424 10.1016/j.devcel.2012.06.001 22898782

[b12] Chatterjee N. Rollins J. Mahowald A. P. Bazinet C. (2011). Neurotransmitter Transporter-Like: a male germline-specific SLC6 transporter required for Drosophila spermiogenesis. PLoS ONE 6, e16275 10.1371/journal.pone.0016275 21298005 PMC3029318

[b13] Dawe H. R. Farr H. Gull K. (2007). Centriole/basal body morphogenesis and migration during ciliogenesis in animal cells. J. Cell Sci. 120, 7–15 10.1242/jcs.03305 17182899

[b14] Delgehyr N. Rangone H. Fu J. Mao G. Tom B. Riparbelli M. G. Callaini G. Glover D. M. (2012). Klp10A, a microtubule-depolymerizing kinesin-13, cooperates with CP110 to control Drosophila centriole length. Curr. Biol. 22, 502–509 10.1016/j.cub.2012.01.046 22365849

[b15] Eddé B. Rossier J. Le Caer J. P. Desbruyères E. Gros F. Denoulet P. (1990). Posttranslational glutamylation of alpha-tubulin. Science 247, 83–85 10.1126/science.1967194 1967194

[b16] Enjolras C. Thomas J. Chhin B. Cortier E. Duteyrat J. L. Soulavie F. Kernan M. J. Laurençon A. Durand B. (2012). Drosophila chibby is required for basal body formation and ciliogenesis but not for Wg signaling. J. Cell Biol. 197, 313–325 10.1083/jcb.201109148 22508513 PMC3328381

[b17] Fabrizio J. J. Hime G. Lemmon S. K. Bazinet C. (1998). Genetic dissection of sperm individualization in Drosophila melanogaster. Development 125, 1833–1843. 9550716 10.1242/dev.125.10.1833

[b18] Gogendeau D. Basto R. (2010). Centrioles in flies: the exception to the rule? Semin. Cell Dev. Biol. 21, 163–173 10.1016/j.semcdb.2009.07.001 19596460

[b19] Gong Z. Son W. Chung Y. D. Kim J. Shin D. W. McClung C. A. Lee Y. Lee H. W. Chang D. J. Kaang B. K. (2004). Two interdependent TRPV channel subunits, inactive and Nanchung, mediate hearing in Drosophila. J. Neurosci. 24, 9059–9066 10.1523/JNEUROSCI.1645-04.2004 15483124 PMC6730075

[b20] Greene J. C. Whitworth A. J. Kuo I. Andrews L. A. Feany M. B. Pallanck L. J. (2003). Mitochondrial pathology and apoptotic muscle degeneration in Drosophila parkin mutants. Proc. Natl. Acad. Sci. USA 100, 4078–4083 10.1073/pnas.0737556100 12642658 PMC153051

[b21] Hicks J. L. Deng W. M. Rogat A. D. Miller K. G. Bownes M. (1999). Class VI unconventional myosin is required for spermatogenesis in Drosophila. Mol. Biol. Cell 10, 4341–4353 10.1091/mbc.10.12.4341 10588662 PMC25762

[b22] Hodges M. E. Wickstead B. Gull K. Langdale J. A. (2011). Conservation of ciliary proteins in plants with no cilia. BMC Plant Biol. 11, 185 10.1186/1471-2229-11-185 22208660 PMC3268115

[b23] Hoyle H. D. Turner F. R. Raff E. C. (2008). Axoneme-dependent tubulin modifications in singlet microtubules of the Drosophila sperm tail. Cell Motil. Cytoskeleton 65, 295–313 10.1002/cm.20261 18205200

[b24] Huang J. Zhou W. Watson A. M. Jan Y. N. Hong Y. (2008). Efficient ends-out gene targeting in Drosophila. Genetics 180, 703–707 10.1534/genetics.108.090563 18757917 PMC2535722

[b25] Ishikawa H. Thompson J. Yates J. R. 3rd Marshall W. F. (2012). Proteomic analysis of mammalian primary cilia. Curr. Biol. 22, 414–419 10.1016/j.cub.2012.01.031 22326026 PMC3298568

[b26] Janke C. Bulinski J. C. (2011). Post-translational regulation of the microtubule cytoskeleton: mechanisms and functions. Nat. Rev. Mol. Cell Biol. 12, 773–786 10.1038/nrm3227 22086369

[b27] Kaplan Y. Gibbs-Bar L. Kalifa Y. Feinstein-Rotkopf Y. Arama E. (2010). Gradients of a ubiquitin E3 ligase inhibitor and a caspase inhibitor determine differentiation or death in spermatids. Dev. Cell 19, 160–173 10.1016/j.devcel.2010.06.009 20643358

[b28] Kavlie R. G. Kernan M. J. Eberl D. F. (2010). Hearing in Drosophila requires TilB, a conserved protein associated with ciliary motility. Genetics 185, 177–188 10.1534/genetics.110.114009 20215474 PMC2870953

[b29] Keller L. C. Romijn E. P. Zamora I. Yates J. R. 3rd Marshall W. F. (2005). Proteomic analysis of isolated chlamydomonas centrioles reveals orthologs of ciliary-disease genes. Curr. Biol. 15, 1090–1098 10.1016/j.cub.2005.05.024 15964273

[b30] Kernan M. Zuker C. (1995). Genetic approaches to mechanosensory transduction. Curr. Opin. Neurobiol. 5, 443–448 10.1016/0959-4388(95)80003-4 7488844

[b31] Kernan M. Cowan D. Zuker C. (1994). Genetic dissection of mechanosensory transduction: mechanoreception-defective mutations of Drosophila. Neuron 12, 1195–1206 10.1016/0896-6273(94)90437-5 8011334

[b32] Kim S. Zaghloul N. A. Bubenshchikova E. Oh E. C. Rankin S. Katsanis N. Obara T. Tsiokas L. (2011). Nde1-mediated inhibition of ciliogenesis affects cell cycle re-entry. Nat. Cell Biol. 13, 351–360 10.1038/ncb2183 21394081 PMC3077088

[b33] Ko H. W. Norman R. X. Tran J. Fuller K. P. Fukuda M. Eggenschwiler J. T. (2010). Broad-minded links cell cycle-related kinase to cilia assembly and hedgehog signal transduction. Dev. Cell 18, 237–247 10.1016/j.devcel.2009.12.014 20159594 PMC2830714

[b34] Laligné C. Klotz C. de Loubresse N. G. Lemullois M. Hori M. Laurent F. X. Papon J. F. Louis B. Cohen J. Koll F. (2010). Bug22p, a conserved centrosomal/ciliary protein also present in higher plants, is required for an effective ciliary stroke in Paramecium. Eukaryot. Cell 9, 645–655 10.1128/EC.00368-09 20118210 PMC2863418

[b35] Li J. B. Gerdes J. M. Haycraft C. J. Fan Y. Teslovich T. M. May-Simera H. Li H. Blacque O. E. Li L. Leitch C. C. (2004). Comparative genomics identifies a flagellar and basal body proteome that includes the BBS5 human disease gene. Cell 117, 541–552 10.1016/S0092-8674(04)00450-7 15137946

[b36] Lindsley K. T. D. (1980). Spermatogenesis. The Genetics and Biology of Drosophila, Vol. 2b (ed. Ashburner M and Wright T R F).London: Academic Press.

[b37] Luo L. Liao Y. J. Jan L. Y. Jan Y. N. (1994). Distinct morphogenetic functions of similar small GTPases: Drosophila Drac1 is involved in axonal outgrowth and myoblast fusion. Genes Dev. 8, 1787–1802 10.1101/gad.8.15.1787 7958857

[b38] Martinez-Campos M. Basto R. Baker J. Kernan M. Raff J. W. (2004). The Drosophila pericentrin-like protein is essential for cilia/flagella function, but appears to be dispensable for mitosis. J. Cell Biol. 165, 673–683 10.1083/jcb.200402130 15184400 PMC2172389

[b39] Mottier-Pavie V. Megraw T. L. (2009). Drosophila bld10 is a centriolar protein that regulates centriole, basal body, and motile cilium assembly. Mol. Biol. Cell 20, 2605–2614 10.1091/mbc.E08-11-1115 19321663 PMC2682601

[b40] Noguchi T. Miller K. G. (2003). A role for actin dynamics in individualization during spermatogenesis in Drosophila melanogaster. Development 130, 1805–1816 10.1242/dev.00406 12642486

[b41] Noguchi T. Lenartowska M. Miller K. G. (2006). Myosin VI stabilizes an actin network during Drosophila spermatid individualization. Mol. Biol. Cell 17, 2559–2571 10.1091/mbc.E06-01-0031 16571671 PMC1474903

[b42] Ostrowski L. E. Blackburn K. Radde K. M. Moyer M. B. Schlatzer D. M. Moseley A. Boucher R. C. (2002). A proteomic analysis of human cilia: identification of novel components. Mol. Cell. Proteomics 1, 451–465 10.1074/mcp.M200037-MCP200 12169685

[b43] Pazour G. J. Agrin N. Leszyk J. Witman G. B. (2005). Proteomic analysis of a eukaryotic cilium. J. Cell Biol. 170, 103–113 10.1083/jcb.200504008 15998802 PMC2171396

[b44] Peel N. Stevens N. R. Basto R. Raff J. W. (2007). Overexpressing centriole-replication proteins *in vivo* induces centriole overduplication and de novo formation. Curr. Biol. 17, 834–843 10.1016/j.cub.2007.04.036 17475495 PMC1885955

[b45] Pisano C. Bonaccorsi S. Gatti M. (1993). The kl-3 loop of the Y chromosome of Drosophila melanogaster binds a tektin-like protein. Genetics 133, 569–579. 8454204 10.1093/genetics/133.3.569 PMC1205344

[b46] Rathke C. Barckmann B. Burkhard S. Jayaramaiah-Raja S. Roote J. Renkawitz-Pohl R. (2010). Distinct functions of Mst77F and protamines in nuclear shaping and chromatin condensation during Drosophila spermiogenesis. Eur. J. Cell Biol. 89, 326–338 10.1016/j.ejcb.2009.09.001 20138392

[b47] Redeker V. Levilliers N. Schmitter J. M. Le Caer J. P. Rossier J. Adoutte A. Bré M. H. (1994). Polyglycylation of tubulin: a posttranslational modification in axonemal microtubules. Science 266, 1688–1691 10.1126/science.7992051 7992051

[b48] Riparbelli M. G. Callaini G. (2007). The Drosophila parkin homologue is required for normal mitochondrial dynamics during spermiogenesis. Dev. Biol. 303, 108–120 10.1016/j.ydbio.2006.10.038 17123504

[b49] Riparbelli M. G. Callaini G. (2011). Male gametogenesis without centrioles. Dev. Biol. 349, 427–439 10.1016/j.ydbio.2010.10.021 20974123

[b50] Riparbelli M. G. Callaini G. Megraw T. L. (2012). Assembly and persistence of primary cilia in dividing Drosophila spermatocytes. Dev. Cell 23, 425–432 10.1016/j.devcel.2012.05.024 22898783 PMC3422508

[b51] Riparbelli M. G. Cabrera O. A. Callaini G. Megraw T. L. (2013). Unique properties of Drosophila spermatocyte primary cilia. Biol. Open 2, 1137–1147 10.1242/bio.20135355 24244850 PMC3828760

[b52] Rodrigues-Martins A. Bettencourt-Dias M. Riparbelli M. Ferreira C. Ferreira I. Callaini G. Glover D. M. (2007). DSAS-6 organizes a tube-like centriole precursor, and its absence suggests modularity in centriole assembly. Curr. Biol. 17, 1465–1472 10.1016/j.cub.2007.07.034 17689959

[b53] Rogowski K. Juge F. van Dijk J. Wloga D. Strub J. M. Levilliers N. Thomas D. Bré M. H. Van Dorsselaer A. Gaertig J. (2009). Evolutionary divergence of enzymatic mechanisms for posttranslational polyglycylation. Cell 137, 1076–1087 10.1016/j.cell.2009.05.020 19524510

[b54] Sabino D. Brown N. H. Basto R. (2011). Drosophila Ajuba is not an Aurora-A activator but is required to maintain Aurora-A at the centrosome. J. Cell Sci. 124, 1156–1166 10.1242/jcs.076711 21402878 PMC3056608

[b55] Seroude L. Brummel T. Kapahi P. Benzer S. (2002). Spatio-temporal analysis of gene expression during aging in Drosophila melanogaster. Aging Cell 1, 47–56 10.1046/j.1474-9728.2002.00007.x 12882353

[b56] Sharma Y. Cheung U. Larsen E. W. Eberl D. F. (2002). PPTGAL, a convenient Gal4 P-element vector for testing expression of enhancer fragments in drosophila. Genesis 34, 115–118 10.1002/gene.10127 12324963 PMC1805626

[b57] Smith J. C. Northey J. G. Garg J. Pearlman R. E. Siu K. W. (2005). Robust method for proteome analysis by MS/MS using an entire translated genome: demonstration on the ciliome of Tetrahymena thermophila. J. Proteome Res. 4, 909–919 10.1021/pr050013h 15952738

[b58] Stolc V. Samanta M. P. Tongprasit W. Marshall W. F. (2005). Genome-wide transcriptional analysis of flagellar regeneration in Chlamydomonas reinhardtii identifies orthologs of ciliary disease genes. Proc. Natl. Acad. Sci. USA 102, 3703–3707 10.1073/pnas.0408358102 15738400 PMC553310

[b59] Tam L. W. Wilson N. F. Lefebvre P. A. (2007). A CDK-related kinase regulates the length and assembly of flagella in Chlamydomonas. J. Cell Biol. 176, 819–829 10.1083/jcb.200610022 17353359 PMC2064056

[b60] Tates A. D. (1971). *Cytodifferentiation during Spermatogenesis in Drosophila melanogaster*. PhD dissertation, University of Leiden, The Netherlands

[b61] Texada M. J. Simonette R. A. Johnson C. B. Deery W. J. Beckingham K. M. (2008). Yuri gagarin is required for actin, tubulin and basal body functions in Drosophila spermatogenesis. J. Cell Sci. 121, 1926–1936 10.1242/jcs.026559 18477609

[b62] Tokuyasu K. T. Peacock W. J. Hardy R. W. (1972a). Dynamics of spermiogenesis in Drosophila melanogaster. I. Individualization process. Z. Zellforsch. Mikrosk. Anat. 124, 479–506 10.1007/BF00335253 4622067

[b63] Tokuyasu K. T. Peacock W. J. Hardy R. W. (1972b). Dynamics of spermiogenesis in Drosophila melanogaster. II. Coiling process. Z. Zellforsch. Mikrosk. Anat. 127, 492–525 10.1007/BF00306868 4625686

[b64] Varmark H. Llamazares S. Rebollo E. Lange B. Reina J. Schwarz H. Gonzalez C. (2007). Asterless is a centriolar protein required for centrosome function and embryo development in Drosophila. Curr. Biol. 17, 1735–1745 10.1016/j.cub.2007.09.031 17935995

[b65] Zhou X. Fabian L. Bayraktar J. L. Ding H. M. Brill J. A. Chang H. C. (2011). Auxilin is required for formation of Golgi-derived clathrin-coated vesicles during Drosophila spermatogenesis. Development 138, 1111–1120 10.1242/dev.057422 21343365 PMC3203210

