# Supplementary material for: Bug22 influences cilium morphology and the post-translational modification of ciliary microtubules
Source: Biol Open. 2014 Jan 7;3(2):138–51. doi: 10.1242/bio.20146577 (PMC3925317; doi:10.1242/bio.20146577)
Supplement: Supplementary Material [file supp_3_2_138__index.html]

Bug22 influences cilium morphology and the post-translational modification of ciliary microtubules — Bug22 influences cilium morphology and the post-translational modification of ciliary microtubules — Supplementary Material 

# Bug22 influences cilium morphology and the post-translational modification of ciliary microtubules

## bio.20146577 Supplementary Material

**Files in this Data Supplement:**

- Supplementary Material - Teresa Mendes Maia et al. doi: 10.1242/bio.20146577
