# Supplementary material for: Bug22 influences cilium morphology and the post-translational modification of ciliary microtubules
Source: Biol Open. 2014 Jan 7;3(2):138–51. doi: 10.1242/bio.20146577 (PMC3925317; doi:10.1242/bio.20146577)
Supplement: Supplementary Material [file supp_bio.20146577_bio.20146577-s1.pdf]

Biology Open

Supplementary Material

Teresa Mendes Maia et al. doi: 10.1242/bio.20146577

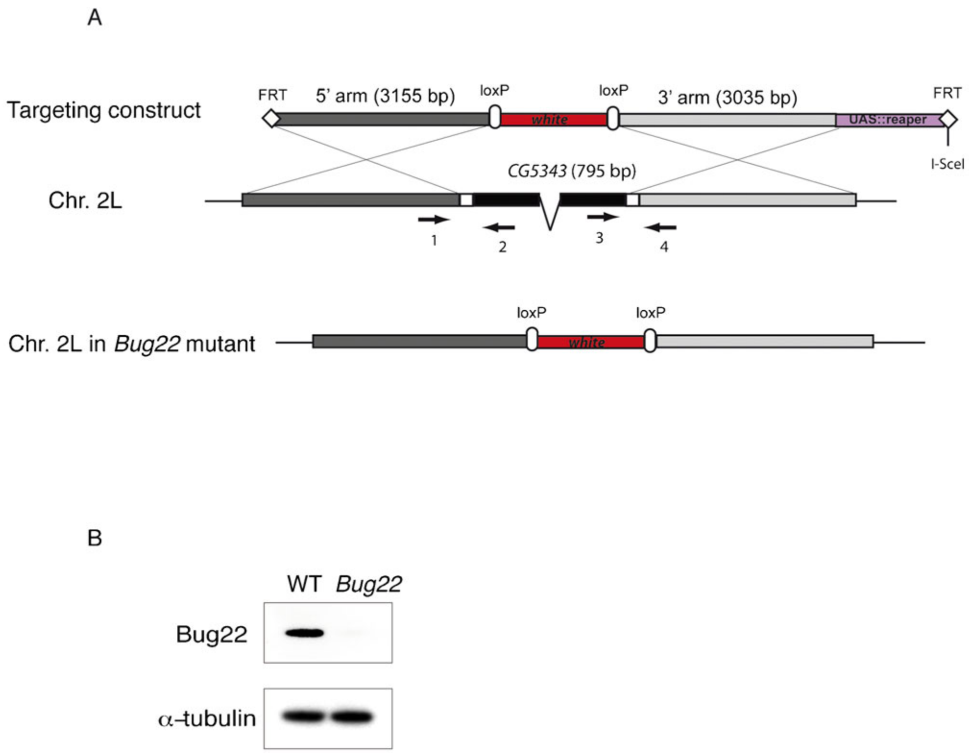

Fig. S1. Generation of a *Bug22* null allele. (A) Strategy followed to knockout *Bug22*. A targeting construct was created (P{$Bug22^{KO}$}) that consisted of an *hsp70::white* (w^+) transformation marker, flanked by two loxP sites and surrounded by two arms of homology: "5′ arm" and "3′ arm", respectively, corresponding to the genomic sequences located immediately upstream and downstream of the *Bug22* locus in the chromosome 2L. Following a strategy described by Huang et al. (Huang et al., 2008), homologous recombination event(s) in the germline of females carrying the P{$Bug22^{KO}$} construct were induced, in order to delete the *CG5343* gene. Recombination events, and thus loss of the junction between Bug22 and its adjacent genes, were tested by PCR using primers 1+2 and 3+4 (see Materials and Methods). (B) Bug22 is detected in WT but not in *Bug22* protein extracts by western blot probed with anti-Bug22 antibodies (GTL3, see Materials and Methods). Anti α-tubulin antibody was used as a loading control.

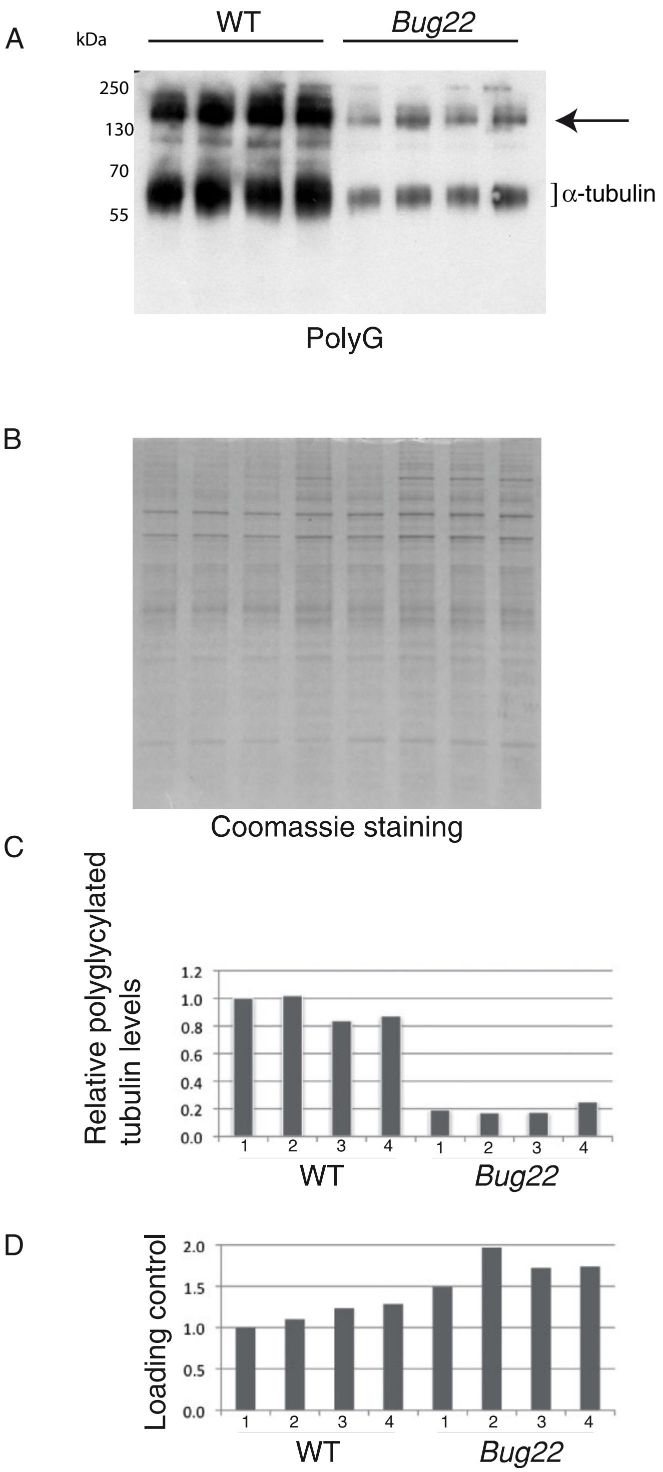

Fig. S2. Quantification of polyglycylation levels in testes. (A) Immunoblots from WT and *Bug22* testes extracts probed with antibodies that recognise polyglycylation (polyG). Samples from four WT and *Bug22* individuals are shown in each immunoblot. (B) Coomassie staining for analysis of total protein levels is shown. The graphs show the quantification of each lane from immunoblots (C) and from the corresponding protein loadings (D).

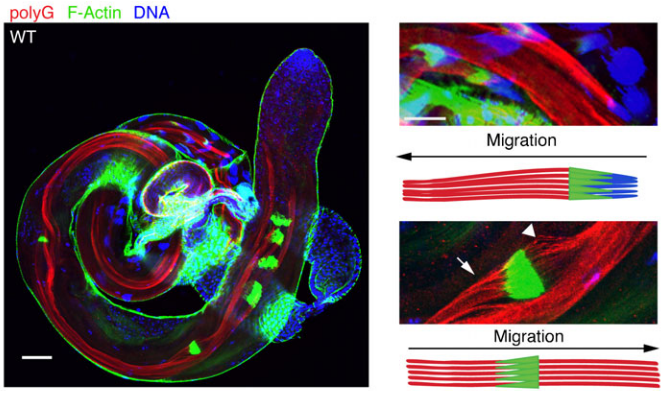

Fig. S3. Sperm polyglycylation takes place before IC migration. Immunostaining of whole mount WT testis stained for PolyG antibodies (shown in red) and F-actin (shown in green) showing that ICs assemble near the sperm nucleus during the needle stages (top inset) and that during IC migration (bottom inset) tubulin polyglycylation appears already evenly distributed along the front (arrowhead) and rear (arrow). Scale bars: 50 µm (low magnification) 20 µm (high magnification).

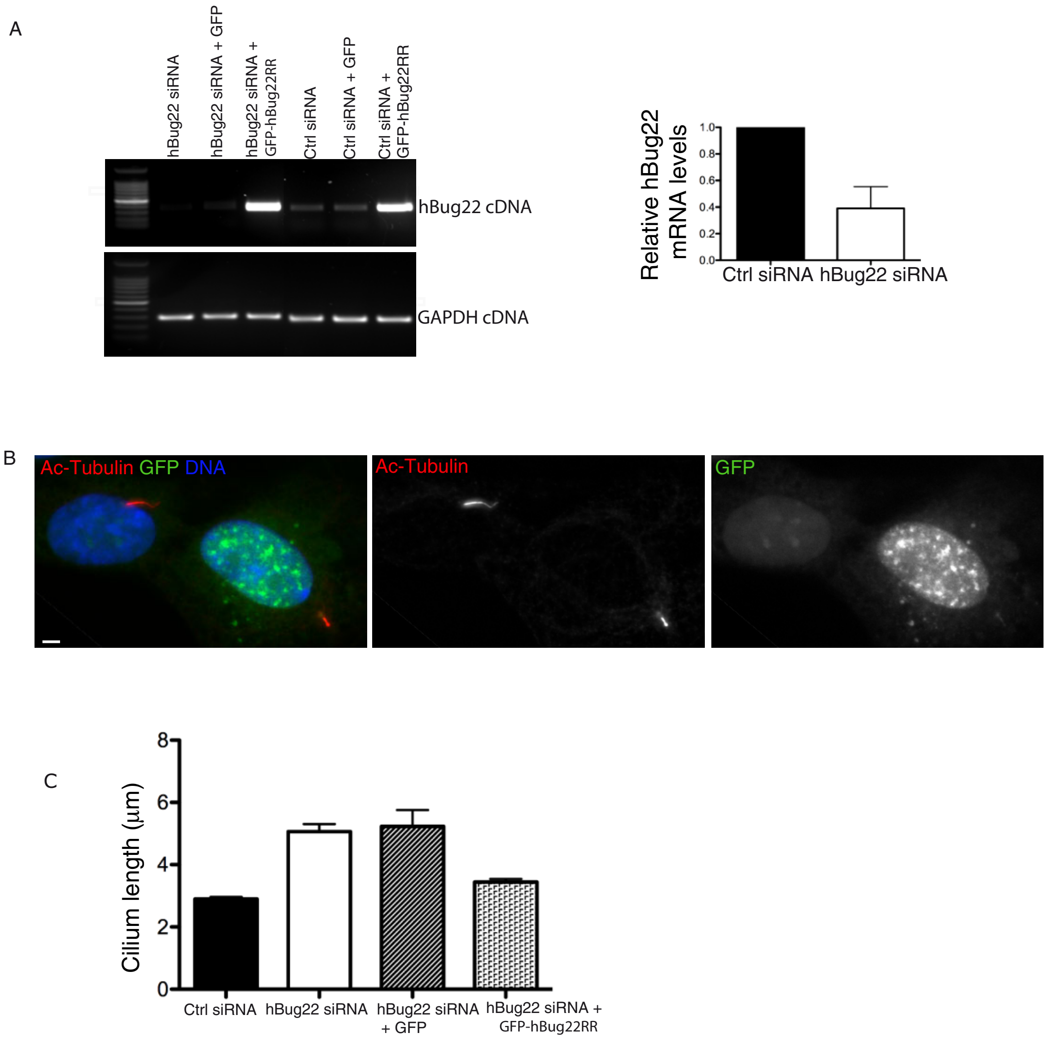

Fig. S4. Control experiments showing specificity of hBug22 depletion. (A) Estimation of Bug22 depletion by RT-PCR analysis of human *Bug22* transcript levels in RPE1 cells co-transfected with negative control (Ctrl) or hBug22 siRNAs + no plasmid/empty pEGFP/pEGP-hBug22RR ("RR" standing for RNAi resistant). *GAPDH* was used as loading control. The graph bars show average ± SEM from three independent experiments. (B) Representative images of hBug22 siRNA treated RPE1 cells, co-transfected with pEGFP-hBug22RR, stained with antibodies that recognise Ac-Tubulin (shown in red) and DNA (shown in blue). The cell positive for pEGFP-hBug22RR (expressing GFP-shown in green) presents normal cilia size, demonstrating that cilia length can be rescued to normal size specifically in cells expressing GFP-hBug22 RNAi resistant transgene. Scale bar: 5 µm. (C) Analysis of cilium length under different transfection conditions. Bars show average ± SEM values from three independent experiments (n>20 per experiment).
